# High Resistance to Quinclorac in Multiple-Resistant *Echinochloa colona* Associated with Elevated Stress Tolerance Gene Expression and Enriched Xenobiotic Detoxification Pathway

**DOI:** 10.3390/genes13030515

**Published:** 2022-03-15

**Authors:** Gulab Rangani, Christopher E. Rouse, Christopher Saski, Rooksana E. Noorai, Vijay Shankar, Amy L. Lawton-Rauh, Isabel S. Werle, Nilda Roma-Burgos

**Affiliations:** 1 Department of Crop, Soil, and Environmental Sciences, University of Arkansas, Fayetteville, AR 72704, USA; crouse579@gmail.com (C.E.R.); iswerle@uark.edu (I.S.W.); 2 Department of Plant and Environmental Sciences, Clemson University, Clemson, SC 29634, USA; saski@clemson.edu; 3 Genomics and Bioinformatics Facility, Clemson University, Clemson, SC 29634, USA; rooksan@clemson.edu; 4 Center for Human Genetics, Clemson University, Greenwood, SC 29646, USA; vshanka@clemson.edu; 5 Department of Genetics and Biochemistry, Clemson University, Clemson, SC 29634, USA; amylr@clemson.edu

**Keywords:** *Echinochloa*, quinclorac resistance, abiotic stress, detoxification, non-target-site resistance (NTSR)

## Abstract

*Echinochloa colona* and other species in this genus are a threat to global rice production and food security. Quinclorac, an auxin mimic, is a common herbicide for grass weed control in rice, and *Echinochloa* spp. have evolved resistance to it. The complete mode of quinclorac action and subsequent evolution of resistance is not fully understood. We analyzed the de novo transcriptome of multiple-herbicide-resistant (ECO-R) and herbicide-susceptible genotypes in response to quinclorac. Several biological processes were constitutively upregulated in ECO-R, including carbon metabolism, photosynthesis, and ureide metabolism, indicating improved metabolic efficiency. The transcriptional change in ECO-R following quinclorac treatment indicates an efficient response, with upregulation of trehalose biosynthesis, which is also known for abiotic stress mitigation. Detoxification-related genes were induced in ECO-R, mainly the UDP-glycosyltransferase (UGT) family, most likely enhancing quinclorac metabolism. The transcriptome data also revealed that many antioxidant defense elements were uniquely elevated in ECO-R to protect against the auxin-mediated oxidative stress. We propose that upon quinclorac treatment, ECO-R detoxifies quinclorac utilizing UGT genes, which modify quinclorac using the sufficient supply of UDP-glucose from the elevated trehalose pathway. Thus, we present the first report of upregulation of trehalose synthesis and its association with the herbicide detoxification pathway as an adaptive mechanism to herbicide stress in Echinochloa, resulting in high resistance.

## 1. Introduction

*Echinochloa* spp. include highly diverse weedy members that are distributed globally, posing a threat to upland and lowland crop production systems [1,2]. The genus is composed of about 60 species, some of which are cultivated as millet crops in underdeveloped regions, but the majority are weedy and invasive [3]. While there is significant diversity within the genus, several species, including the dominant *E. colona* (junglerice) and *E. crus-galli* (barnyardgrass), are phenotypically similar [4]. A history of co-domestication and continued selection in rice (*Oryza sativa*) culture have resulted in crop mimic strains within these species [5,6]. In the USA, 13 *Echinochloa* species occur in 48 of the contiguous United States [7]. Of these, the most impactful in agricultural areas, specifically in rice and rice-based cropping systems, include *E. colona, E. crus-galli, E. phyllopogon* (late watergrass), and *E. oryzoides* (early watergrass). A single *E. crus-galli* plant can reduce rice yield by up to 65 kg ha^−1^; it is second only to weedy rice (*Oryza sativa* L.) in terms of impact to production [8,9]. To maximize rice production, weeds must be controlled, as they are the most limiting biotic factor to attaining the crop yield potential [1].

Propanil, a photosystem II inhibitor, was the among first highly effective and selective herbicides for *Echinochloa* control in USA rice production; this was followed by quinclorac, an auxin mimic, and several herbicide chemistries that disrupt fatty acid or amino acid synthesis [10]. Resistance to herbicides in weedy species is an adaptive evolutionary trait selected by repeated exposure to the same herbicide mode/mechanism of action. Two terminologies are used to describe resistance: target-site resistance (TSR) and non-target-site resistance (NTSR). TSR pertains to a modification in a herbicide target enzyme, resulting in reduced binding efficiency or complete exclusion of the herbicide. NTSR encompasses several mechanisms, including physiological, biochemical, and/or structural responses that work via cascading processes, leading to herbicide detoxification, redistribution, or sequestration and eventually reducing the herbicide concentration at the site of action [11,12]. Alternative mechanisms may result in increased neutralization of the destructive/ downstream action of the herbicide (i.e., increased antioxidant activity), even if the concentration of herbicide reaching the target site remains the same. NTSR mechanisms are the least understood and most problematic, as they may result in cross-resistance to other herbicides, including those not used previously or yet to be discovered. Modifications toward NTSR, by nature, could also enhance abiotic stress tolerance, which, in turn, enhances weediness. The resistance of *Echinochloa* species to herbicides involves both mechanisms: TSR to multiple amino acid synthesis inhibitors [13,14] and photosystem II inhibitors [15] and NTSR to amino acid synthesis inhibitors, including clomazone, propanil [16], and quinclorac [17]. In dicots, quinclorac (like other auxin mimics, i.e., dicamba or 2,4-D) disrupts auxin regulation, causing elevated ethylene and abscisic acid (ABA) production, which results in uncontrolled cell elongation and growth and ultimately leads to plant death [18]. In grass species, the mode of action of quinclorac has been suggested to involve the induction of cyanide to toxic levels that result as a byproduct of excessive ethylene biosynthesis in response to quinclorac [19,20,21].

Attempts to understand the mechanisms of herbicide resistance in weedy species has been limited to monogenic- or single-trait response characterization. This is due to both a limitation in resources to investigate the molecular response and a lack of understanding of the role of these responses toward resistance evolution. Evolution occurs through adaptive responses that modify existing biological pathways and the underlying cellular processes. Understanding these mechanisms occurring in weedy plants adapted to herbicide selection pressure is important for designing novel strategies for weed control.

Arkansas is the largest producer of rice in the United States. Owing to the long history of intensive herbicide selection pressure in Arkansas rice agriculture, the probability of dynamic physiological adaptation to herbicide-induced stress in *Echinochloa* spp. is high. Practically, *Echinochloa* spp. in Arkansas have evolved resistance to all major herbicides currently used in rice production [22]. The multiple-resistant *E. colona* population, ECO-R, represents a type of population among a diverse mix of resistant populations from Arkansas, characterized by its extremely high resistance level to quinclorac and propanil [23]. A comprehensive investigation of the physiological basis for multiple resistance revealed that ECO-R harbors a resistance mechanism different from what is known about quinclorac-resistant species [23]. The known cytochrome P450 inhibitors did not reverse the resistance to quinclorac in ECO-R, although this finding cannot completely exclude herbicide detoxification by cytochrome P450 because the tested inhibitor may not inhibit all other relevant P450 genes. The absorption or translocation of quinclorac between ECO-S and ECO-R did not differ. This information on ECO-R indicates that high resistance is driven by NTSR mechanism(s) that are not yet deciphered. A comprehensive approach to identify NTSR mechanisms would be comparative transcriptome sequencing of the resistant and sensitive genotypes. In this study, we investigated the possible resistance mechanisms in ECO-R using high-throughput RNA-seq technology. This study identified the genes and gene networks responding to the quinclorac herbicide in susceptible and resistant genotypes over 24 h of herbicide action. We identified the physiological pathways that are involved in resistance to quinclorac and the response to abiotic stress. The differentially expressed genes indicate that high resistance to quinclorac is endowed by upregulation of trehalose biosynthesis with specific glycosyltransferase genes to conjugate the quinclorac molecule with UDP-glucose following herbicide treatment.

## 2. Results

### 2.1. RNA Sequencing, De Novo Assembly, and Functional Annotation of the E. colona Transcriptome

**Figure 1:**
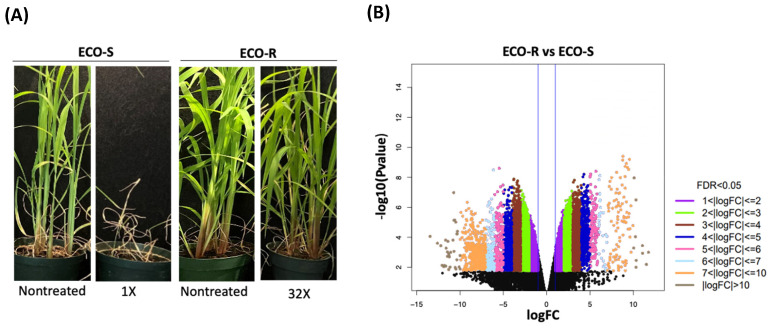

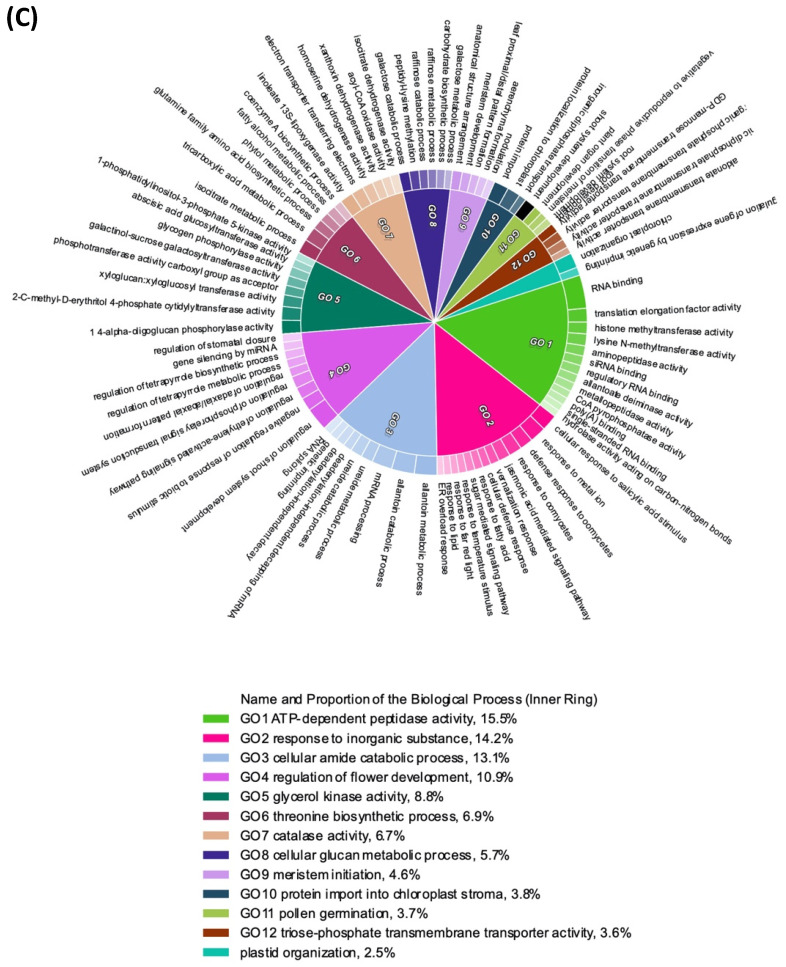
Phenotype and transcriptome analysis of quinclorac-resistant *Echinochloa colona* (ECO-R). (**A**) Response to quinclorac 21 d after treatment. Photographs of the susceptible genotype ECO-S (left), nontreated and treated (1X field dose), and the resistant genotype ECO-R (right), nontreated and treated (32X field dose). (**B**) Volcano plot depicting the differentially expressed genes in the nontreated ECO-R sample compared to ECO-S. The *p* value (−log base 10) for differential gene expression is plotted on the Y-axis. The colored dots on the left indicate genes with significantly downregulated expression and the colored dots on the right indicate genes with significantly upregulated expression. (**C**) CirGO (circular gene ontology) visualization of GO terms enriched in differentially expressed genes in nontreated ECO-R vs ECO-S (*p* < 0.05).

ECO-R is highly resistant to quinclorac herbicide (Figure 1A) [23]. In order to explore the mechanism of resistance to quinclorac, we performed RNA sequencing of ECO-S and ECO-R genotypes before (nontreated) and 24 h after quinclorac treatment (1X, recommended field dose). The transcriptome was assembled from 545,000,000 raw read pairs, which generated over 109,000 transcripts (Table S1). An analysis of conserved plant ortholog sequences (BUSCO) revealed that approximately 75% of the transcriptome was resolved. A functional annotation revealed 60,530 genes retained, which were used to characterize the transcriptome. Homology to other organisms was as expected, given the parameter of the annotation. However, the sequence homology to *Oryza sativa* var. *japonica* (17.7%) is of value, given the early co-domestication of these species and their co-evolution throughout the history of rice production [5,6].

### 2.2. Identification of Differential Transcriptome between the ECO-R and ECO-S Genotypes

Expression profiles of the nontreated ECO-R and ECO-S *Echinochloa* genotypes were compared to identify differences in constitutive gene expression. Constitutive gene expression differed by 19,478 genes between ECO-R and ECO-S (Figure 1B), 99% of which were annotated (Tables S2 and S3)**.** The gene ontology (GO) term analysis was conducted to identify the function of the differentially expressed genes (DEGs) (>two-fold with *p* < 0.05) in nontreated ECO-R compared to ECO-S. Overall, the gene ontology analysis yielded 83 biological process, 19 cellular component, and 42 molecular function category terms that were enriched in ECO-R relative to ECO-S without quinclorac treatment (Figure 1C, Table S4). The superclusters of terms such as allantoin metabolic process, ureide metabolic process, glucan biosynthetic process, raffinose catabolic process, phytol metabolic process, cellular defense response, regulation by genetic imprinting, and many other biological processes were enriched in ECO-R. The elevated expression of differentially expressed genes of these biological processes is noteworthy for the fact that these plants were not grown under biotic nor abiotic stress. The role of raffinose sugars is known in stress tolerance mechanisms, particularly as osmo-protectants. They also function as a carbon reserve and for signal transduction [24,25,26,27]. Ureides allantoin and allantoin acid are known to serve as long-distance N transport forms in nodulating plants [28]. Recently, its role has been addressed in the source-to-sink transport of photoassimilates in non-nodulating plants [29]. Genes associated with growth and development, such as carbon metabolism and photosynthesis, were constitutively enriched in ECO-R over ECO-S, and, thus, suggest the role of ureides allantoin in meeting the demand and utilization of products from carbon assimilation and photosynthesis. The expression of sugar transporters was also higher in ECO-R, which is reasonable, given the indication of increased sugar synthesis. Several putative ethylene-responsive transcription factor (ERF) genes were constitutively expressed (Table S4), indicating heightened transcriptional activity to effect ethylene-mediated responses. The majority of these ERFs, both repressors and activators, bind to the GCC-box containing cis-acting elements that are linked to disease-, cold-, salt-, and/ or water-deprivation stress tolerance [30,31].

Several genes within detoxification gene families were constitutively upregulated in ECO-R relative to ECO-S (Table S4). The cytochrome P450, glutathione-S-transferase (GST), and glycosyltransferase (UGT) enzymes were elevated in ECO-R, indicating possible involvement in intrinsic stress tolerance in ECO-R and the regulation of cellular homeostasis. Several aminotransferase-, amylase-, hydrolase-, and peptidase enzymes were also expressed, indicating active modifications of biomolecules. Since glycosyltransferases are known to be involved in natural growth processes and detoxification pathways, it is possible that some of these enzymes might have evolved to respond to endogenous metabolites under natural conditions as well xenobiotics, such as quinclorac, caused by repeated exposure to the herbicide.

### 2.3. Identification of the Quinclorac-Induced Transcriptome in the ECO-S and ECO-R Genotypes after 24 h

**Figure 2:**
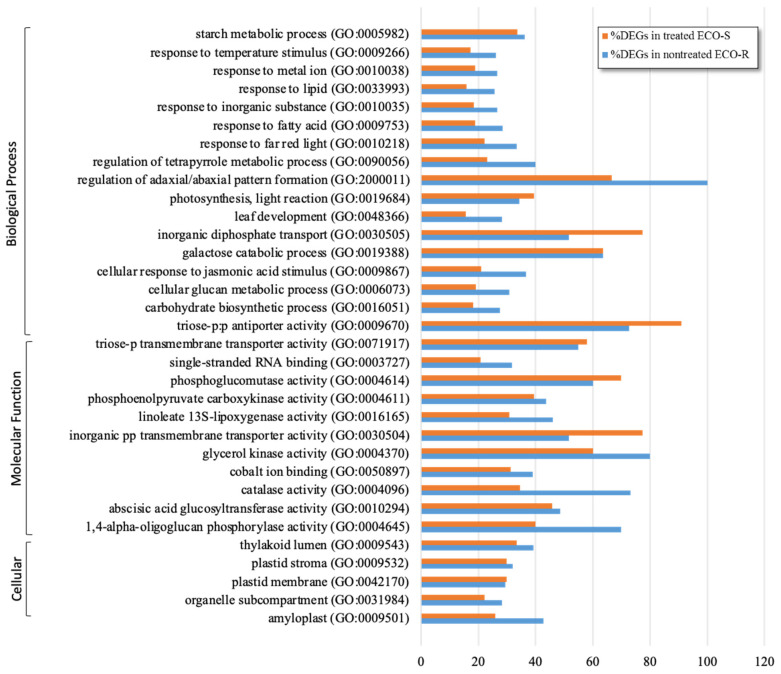
Enrichment of similar GO terms between nontreated quinclorac-resistant (ECO-R) and treated quinclorac-susceptible (ECO-S) *Echinochloa colona* genotypes. The percentages of differentially expressed genes within each GO term are presented.

By comparing the transcriptomes of quinclorac-treated with nontreated plants of the ECO-S genotype, several GO terms with overlapping functions in multiple biotic and abiotic stresses were found to be enriched upon quinclorac treatment. The ethylene-activated signaling pathway and the abscisic-acid-activated signaling pathway were enriched, both of which would be a direct response to the herbicide, based on the literature. A total of 268 biological processes were enriched in response to quinclorac in the ECO-S genotype (Table S5), indicating a broad response, characteristic of auxin action. These gene families are not only involved in plant maintenance but also in the general plant stress response, indicating the utilization of energy-consuming coping mechanisms against herbicide stress in the ECO-S genotype, though none of them were effective in neutralizing or avoiding quinclorac phytotoxicity. Many enriched GO terms in response to quinclorac in ECO-S were similar to those observed in the constitutively upregulated transcriptome of ECO-R (without quinclorac) (Figure 2). Although similar GO terms were enriched, the number of DEGs in nontreated ECO-R were higher than in ECO-S, indicating that ECO-R already has a stronger built-in stress-coping mechanism than ECO-S.

The deduced signal cascades for ECO-S and ECO-R after quinclorac treatment are depicted in supplementary Figure S1. In ECO-S, the auxin characteristic response was found to be triggered by the induction of putative 1-aminocyclopropane 1-carboxylic acid (ACC) oxidase enzymes 24 h after treatment, which may lead to an increase in ethylene and toxic cyanide concentrations (Table S6), based on the literature [19,20,21]. The expression of putative *ACC synthase* was downregulated after 24 h, suggesting that the elevated ethylene concentration might have exerted a negative feedback inhibition of the ethylene synthesis pathway in ECO-S. A concomitant induction of the 9-cis-epoxycarotenoid dioxygenase (NCED) enzyme would promote abscisic acid (ABA) production. The induction of many isoforms of ABA 8′-hydroxylase were enriched, possibly to limit the concentration of ABA. Increased ABA synthesis would result in the closing of stomata, limiting water movement and gas exchange, but would also lead to the buildup of reactive oxygen species that cause tissue decay and senescence [32]. Thus, the stimulatory effects of the exogeneous auxin (quinclorac) and ABA responses were enriched in ECO-S. This deduction was supported by several enriched processes related to anaerobic conditions in ECO-S (Table S5). Collectively, these genes drive the physiological response of ECO-S (sensitive *E. colona* populations in general) to quinclorac.

The transcriptome of ECO-R, upon quinclorac treatment, showed a heightened ethylene response (Table S6). Many isoforms of putative *ACC oxidase* were enriched, suggesting more ethylene production in ECO-R compared to ECO-S. A significant upregulation of multiple isoforms of *ET-stabilized transcription factors* (*EIN3/EIL1)*, which are master regulators of ethylene-responsive genes, suggests the tailored regulation of ethylene signaling in ECO-R [33,34]. Moreover, putative nitrilase and nitrilase dehydratase, which are part of the β-cyanoalanine synthase (β-CAS) pathway, were found to be upregulated in ECO-R after quinclorac application compared to ECO-S, indicating active degradation of the cyanide generated by ethylene synthesis [35]. The *ABA 8*′-*hydroxylase* genes were present at low levels, with downregulation of NCED in ECO-R compared to ECO-S upon treatment. This pattern indicates that no significant induction of NCED was required for ABA synthesis and/or that the ABA concentrations were not high enough to warrant the production of more hydroxylase enzyme (Figure S1, Table S6). Reductions in ABA synthesis and reduced signal perception may reduce the effects of quinclorac in ECO-R, considering its pivotal role in quinclorac-mediated toxicity. The upregulation of ascorbate recycling and the glutathiol- and thioredoxin-linked system indicates efficient ROS scavenging activities in ECO-R compared to ECO-S after quinclorac treatment (Table S7). Many of the putative stress-responsive genes that encode proline metabolic enzymes, such as ornithine-γ- aminotransferase (OAT) and pyrroline-5-carboxylate synthetase (P5CS), were found to be upregulated in ECO-R and were absent in ECO-S upon quinclorac treatment. The upregulation of these functional genes would indicate proline accumulation, which has an osmoprotection role under abiotic stress conditions. Although a biochemical assay is needed to confirm it, the enrichment of the glutamate synthesis GO term in ECO-R is indicative of the homeostasis of proline accumulation [36,37].

**Figure 3:**
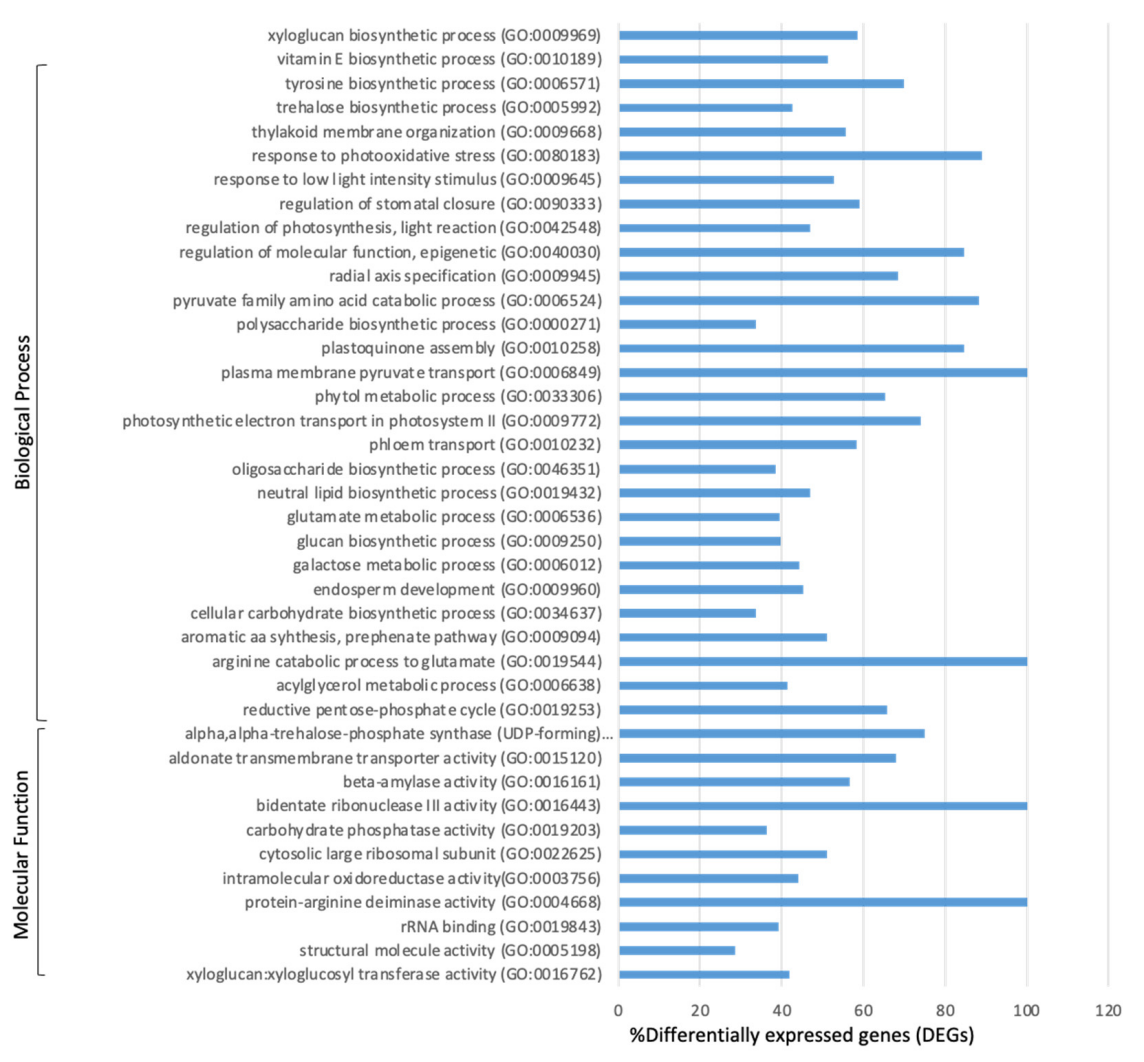
Enrichment of GO terms in quinclorac-resistant *Echinochloa colona* (ECO-R) after quinclorac treatment. This enrichment response in ECO-R is relative to the treated susceptible genotype (ECO-S) transcriptome, and the percentages of differentially expressed genes within each GO term are presented.

Apart from the direct response to auxin observed in ECO-R, many ontological terms that were absent in ECO-S, including trehalose biosynthesis, the arginine catabolic process to glutamate, the xyloglucan biosynthetic process, and the phytol metabolic process (Figure 3, Table S7) were enriched in ECO-R, indicating its probable function in coping with the herbicidal stress without lethal or adverse effects. Many genes were differentially upregulated in ECO-R, including ureide permease (UPS), raffinose synthase, and rhamnose synthase (Table S7).

### 2.4. Identification of Xenobiotic Detoxification Genes following Quinclorac Treatment in ECO-R

A greater number of xenobiotic detoxification candidate genes were induced in treated ECO-R than in ECO-S (Table 1), indicating an evolution of this genotype in terms of detoxification capacity. Many cytochrome (CYP) P450 family genes, such as *CYP709B1*, *CYP709B2*, *CYP72A14*, *CYP72A15* and *CYP72C1,* were unique among the cytochrome response upon the quinclorac treatment in ECO-R. These CYP genes are abiotic stress responsive, and their early induction upon quinclorac treatment indicates their potential involvement in phase I chemical degradation. UGT73D1 with quercetin O-activity and UGT75D1 with potential xenobiotic detoxification activity, based on homology, were upregulated in response to quinclorac and were downregulated in ECO-S. UGT75D1 is of great interest as a potential protein enabling resistance, given its high expression in ECO-R and its known activity on environmental toxins and xenobiotics [38]. A few transcripts of UGT73D1 and UGT75D1 were found be constitutively upregulated in nontreated ECO-R and were further induced upon quinclorac treatment (Table 1 and Table S3). A range of ATP-binding cassette (ABC) transporters were upregulated in ECO-R, suggesting the transportation of conjugated auxins in the vacuole [39,40]. Many transcription factors were differentially upregulated in ECO-R, suggesting the control of specific genes upon quinclorac treatment.

### 2.5. Quantitative PCR Validation of Genes Identified by Transcriptome Analysis

**Figure 4:**
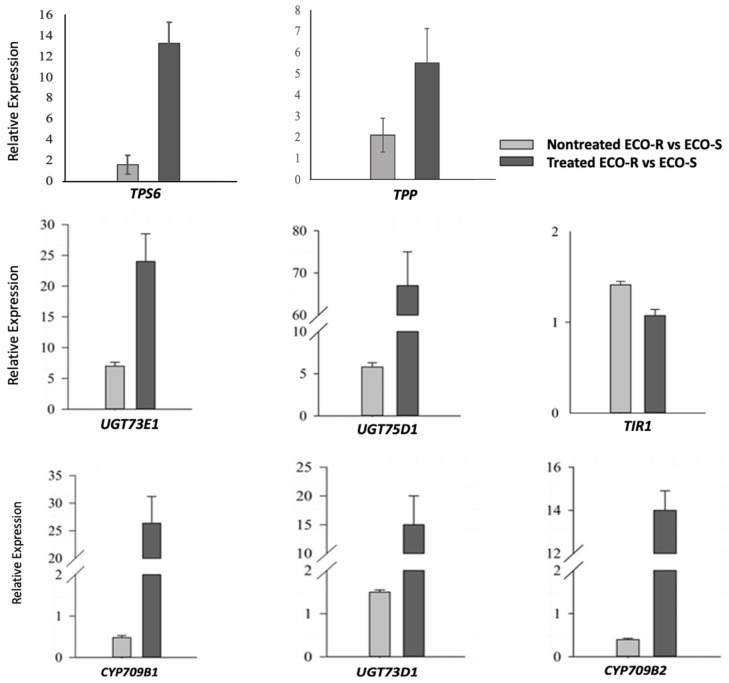
Validation of the expression of eight candidate genes by RT-qPCR.

A subset of selected genes was quantified by real-time quantitative PCR (qPCR) to validate the expression magnitude identified by RNA-seq analysis using both genotypes, ECO-R and ECO-S, and nontreated and treated (24 h) samples (Figure 4). A total of eight genes were evaluated to validate the RNA-seq data. Constitutive expression was measured by comparing nontreated ECO-R versus nontreated ECO-S. The differential induction of gene expression between genotypes upon quinclorac treatment was calculated by comparing treated ECO-R with the treated ECO-S samples. The constitutive induction of *TPP* and *TPS* transcripts ranged from 1.6- to 2.1-fold, whereas, upon quinclorac treatment, it increased to 5.5- to 13.2-fold in ECO-R. We selected a few UGT candidates and checked its expression in ECO-R. *UGT75D1* was constitutively induced 5.8-fold in nontreated ECO-R; an additional, and greater, induction (67-fold) occurred after 24 h in the quinclorac-treated sample compared to quinclorac-treated ECO-S. The expression of *UGT73E1* was constitutively higher in ECO-R, which further increased to 24-fold following quinclorac treatment. The constitutive upregulation of *UGT73E1* in ECO-R was observed with a higher *p*-value (0.07) in RNA-seq data, and, therefore, it is not listed in Table S3. Higher inductions of *CYP709B1*, *CYP709B2, UGT73D1, and UGT75D1* in ECO-R by quinclorac were validated compared to the treated ECO-S sample. *TIR1* was not differentially expressed between the genotypes under nontreated and treated conditions in the transcriptome and the validation test. Altogether, the higher expression of the trehalose genes and detoxification candidates in the qPCR experiment validates the transcriptome data.

### 2.6. Sequence Analysis of Transport Inhibitor Response 1 (TIR1)

It is not yet clear if quinclorac binds to TIR1 or other receptors, but since quinclorac mimics the action of natural auxin, it is proposed that it may bind to TIR1/AFBs to effect further interaction [41]. The putative TIR1 sequence from the ECO-S and ECO-R transcriptomes was retrieved from the transcriptomes. No difference was observed between genotypes, indicating the presence of NTSR mechanism(s) for quinclorac insensitivity in ECO-R rather than a mutation in the auxin-binding domain.

## 3. Discussion

Adaptation is a critical process of weedy plant biology, allowing species to respond to adversity and persist in agricultural landscapes. The evolution of multiple resistance to herbicides used for weed control in crop production is increasing, and weed resistance to herbicides has become a threat to global food security and agricultural sustainability [42]. Thus far, the mechanisms of quinclorac action in resistant *Echinochloa* spp. have been largely described biochemically. To investigate the molecular mechanism and the candidate genes involved in resistance to quinclorac, we compared the transcriptome of resistant and susceptible *Echinochloa* populations using nontreated and treated (24 h) leaf tissues. This comparison allowed for a better understanding of the signal cascade, which, in turn, greatly improved our understanding of how *E. colona* responds and adapts to the auxin-mimic herbicide quinclorac.

The nontreated transcriptome profile of ECO-R provided an insight into its adaptation to withstand the auxinic herbicidal stress. The strong constitutive make-up of ECO-R reflects the accumulation of herbicide-stress-adaptation genes over time. A high abundance of genes associated with carbon assimilation and energy production processes were upregulated in ECO-R compared to ECO-S (Figure 1C) and can therefore be construed as important candidates for controlling the physiology of the resistance mechanism. It can be inferred that a higher concentration of these gene products may permit a higher metabolic flux that increases the rate of biosynthesis or energy production and confers a natural metabolic fitness, which allows a tolerance to subsequent severe stress in the plant following quinclorac treatment. Moreover, the enrichment of similar GO terms between nontreated ECO-R and treated ECO-S (Figure 2) suggests that the critical response to quinclorac became ‘imprinted’ in the genomic memory of ECO-R and was eventually inherited as a constitutive trait of the population. Follow-up functional genomics research may enlighten us on how the coordinated upregulation of some gene networks became constitutively fixed in ECO-R.

The transcriptional response of ECO-R to quinclorac was vastly different from that of ECO-S, demonstrating population divergence under immense herbicide selection pressure. After 24 h, in ECO-R, there was an active ethylene signaling response, together with a strong antioxidant response. ROS homeostasis was evident by the activation of enzymatic and non-enzymatic antioxidant defense (Table S7), suggesting the ECO-R genotype evolved to resist the auxin-mediated oxidative stress. A specific GO category, ‘trehalose biosynthesis’ was over-represented in ECO-R in response to quinclorac. Trehalose biosynthesis has been extensively studied for its role in abiotic stress tolerance [43,44,45,46,47,48] but not in tolerance to herbicides. Trehalose is a unique biological sugar that has been characterized as an important factor in cellular metabolism and is critical for growth and development [49,50]. This nonreducing sugar has several roles of interest for this research: its regulatory and signaling effect on sucrose, its role in membrane stability, and its ability to neutralize reactive oxygen species. The abundance of putative trehalose synthase and trehalose-phosphate phosphatase transcripts in ECO-R (Table S7) implies elevated trehalose content, which may serve active roles in auxinic stress tolerance and aid in averting herbicide phytotoxicity. Upregulation of putative UPS in ECO-R indicates a balanced use of C and N metabolisms in ECO-R upon quinclorac treatment [29]. Recently, rhamnosylation of kaempferol was determined as an endogenous inhibitor of auxin transport [51]. The upregulation of multiple putative rhamnose synthase genes in ECO-R upon quinclorac treatment suggests that rhamnose synthesis may also contribute to quinclorac resistance.

There is limited information about the genes endowing metabolic-based resistance to quinclorac. We previously studied quinclorac metabolism using ECO-R and ECO-S [23], which showed parental quinclorac as the major metabolite in ECO-R after 24 h. A comprehensive study on quinclorac metabolism presented by the Joint FAO/WHO Meeting on Pesticide Residues (JMPR) 2015, available online: https://www.fao.org/fileadmin/templates/agphome/documents/Pests_Pesticides/JMPR/Evaluation2015/QUINCLORAC__287_.pdf (accessed on 28 February 2022), also indicated parental quinclorac as a major metabolite in animals and plants. It was explained in the report that parental quinclorac might have been released from conjugates upon reaction with acid or alkali during extraction. Therefore, it is not clear whether the remaining parent compound also includes the parent molecule released from conjugates. Considering our metabolite data and the afore-mentioned report, we conclude that the majority of absorbed quinclorac is not transformed into another metabolite, but the intact parental molecule is modified through conjugation. We found upregulation of a few candidate detoxifying genes in ECO-R, which were uniquely expressed compared to ECO-S in this study. A large number of genes involved in reactions that utilize carbon, e.g., UDP-glycosyltransferase genes involved in the glycosylation of hormones, metabolites, or toxins, were found to be upregulated in ECO-R upon quinclorac treatment and were also expressed at higher constitutive level in ECO-R (Table 1 and Table S4). The glycosylation of auxins is recognized as an important biochemical modification for auxin homeostasis in plants [38,52,53,54,55,56]. Quinclorac is a structural mimic of an auxin. Therefore, the glycosylation of quinclorac implies a major molecular modification. Our previous work on the physiological assessment of resistance mechanisms in ECO-R also indicated that quinclorac detoxification could be driven largely by glycosyl transferase [23]. UDP-glycosyltransferase enzymes are known to require activated sugars, such as UDP-glucose, which has to be available to conjugate large, foreign, or unnecessary molecules. Therefore, the enrichment of trehalose biosynthesis, which is an energy-buffering pathway (particularly under stress), in ECO-R strongly suggests its association with quinclorac metabolism by providing free UDP-glucose under high demand. The significant upregulation of glucosyltransferase genes upon treatment with quinclorac apparently did not detoxify the herbicide without a corresponding upregulation of UDP-glucose in ECO-S. Similar information was previously reported where enrichment of UGT family genes were selected with the notable upregulation of T6P [57].

Some studies have shown that trehalose helped maintain a rich diversity and high abundance of certain cytochrome P450 family genes [58,59]. Yet another study showed that the addition of trehalose in growth media enhanced the synthesis of the natural product encoded by a cytochrome-P450-rich biosynthetic pathway [60]. Their data indicate that trehalose enhanced the functionality of cytochrome P450s, mainly by reversing the misfolding of nascent polypeptides. Another benefit of trehalose build-up is its potential role in stabilizing cell membranes following herbicide treatment. Membrane stabilization could result from trehalose, serving as a protectant against cyanide-induced membrane decoupling, the production of ROS under high light intensity, or long-term water deprivation stress. The trehalose sugar is capable of forming hydrogen bonds with the hydrophobic head of the lipid bilayer, stabilizing it against oxidative and water deprivation stress, or potentially (in the case of quinclorac) against cyanide decoupling [61,62]. This will also stabilize membranes against destructive compounds, such as free radicals and ROS. More importantly, trehalose has the ability to scavenge both hydrogen peroxide and ROS, reducing the negative effects they may cause following herbicide action [62,63,64]. This would mitigate the destructive secondary or tertiary effects of the herbicide. The selection of trehalose as a principal osmolyte to bestow the protection is highly valued against desiccation as a selective pressure [65]. However, the potential role of trehalose has not been described as a preventative measure against herbicide action nor has it been described in terms of herbicide response. Considering the abundance of evidence on the activities of trehalose under abiotic stress, it is very likely that this compound contributes to NTSR mechanisms to quinclorac and possibly other herbicides. Understanding the specific role of trehalose in stress mitigation is still ongoing research, and ECO-R can serve as a good model for such study.

Overall, we propose that the candidate UGT family genes that were differentially and/or uniquely induced in ECO-R following quinclorac treatment have a role in quinclorac detoxification; specifically, through an interaction with the carboxylic acid side chains. These candidate genes have not been described as a detoxification enzyme for quinclorac, but GT enzymes have been described as NTSR factors involved in phase II of xenobiotic detoxification [11]. Phase II GT activity requires the oxidation or hydrolysis of compounds to expose OH^−^ or NH_2_ for conjugation. The quinclorac molecule contains an exposed OH^−^ side group with which UGTs can interact, suggesting the phase I step would not be necessary. It is known that *Arabidopsis* UGT75D1 binds to IAA but preferentially binds to endogenous kaempferol and exogenous 2,4,5-trichlorophenol, another pesticide [66]. Not much literature is available, but the UGT73D and UGT73C family genes could be functionally related and could have possible quercetin O-activity [67,68]. All these compounds (IAA, quercetin, and kaempferol) contain similar phenolic ring structures, OH^−^ side groups, and exposed chloride groups as quinclorac. Therefore, putative U73C1, U73C2, U73D1, U75D1, and U75D1-like gene products may be able to use quinclorac as a substrate in ECO-R. This reaction would require free UDP-glucose, which UGTs could conjugate to the quinclorac molecule. An instantaneous supply of UDP-glucose due to the upregulation of trehalose synthesis and other sugar synthesis pathways would fulfill this requirement. We proposed quinclorac conjugation involving the interconnected trehalose biosynthesis with the potential endogenous (IAA, quercetin, and kaempferol) and exogenous quinclorac with affinity for UGTs. Thus, our research produced the first illustration of the interplay between elevated trehalose metabolism and the detoxification pathway as a means to survive quinclorac application. This research also addresses important questions about the evolution of a gene regulation network in herbicide-resistant plants. To confirm the role of candidate genes in quinclorac detoxification, a transgenic approach using green foxtail (*Setaria viridis*) or *Arabidopsis* as a model system will be employed in future work. Further, using RNAi or a virus induced gene silencing (VIGS) approach, candidate genes can be manipulated in *Echinochloa* to reverse the resistance as a practical application.

## 4. Conclusions

The response of susceptible *E. colona* to quinclorac conforms to what is described in the literature. In susceptible *E. colona*, quinclorac induces various stress-response and stress-mitigation genes, including xenobiotic detoxification genes, to reduce the impact of the herbicide. This is expected. However, none of these relieved the herbicide phytotoxicity. ECO-R is a genotype with an extremely high level of quinclorac resistance (up to 32X the recommended rate) and it has a significantly different transcriptome profile than the susceptible genotype under 1X quinclorac treatment. The transcriptome of ECO-R indicates the ability to adapt to abiotic stress and a predisposition to tolerate harsh conditions, including herbicide treatment. The enrichment of the trehalose pathway supports its pivotal role in the evolved adaptation mechanisms in this population. Not only would high trehalose concentrations aid in stress response and potentially mitigate the negative effects of the herbicide, but the trehalose pathway enrichment may also facilitate the potential glucosyltransferase-mediated quinclorac detoxification mechanism. Therefore, this *E. colona* genotype appears to have evolved two complementary mechanisms, resulting in extremely high resistance to quinclorac: (a) protection from, or tolerance to, abiotic stress via an enriched trehalose synthesis and (b) the detoxification of quinclorac by glucosyltransferase. Consequently, ECO-R serves an important resource for future investigation on the transcription factors governing the pathways involved in mitigation of abiotic stress.

## 5. Materials and Methods

### 5.1. Plant Materials

*Echinochloa* spp. were collected from rice and soybean production fields in Arkansas, USA between 2010 and 2016 to survey the status of herbicide resistance. Seeds were bulk-sampled from plants that remained in the field at the end of the season. The samples were tested for resistance to various herbicides [22]. Two populations of *E. colona* were selected for this experiment; both were collected in 2010. ECO-R is a multiple-resistant population with extreme resistance to propanil and quinclorac; it is resistant to >32X the field dose of the latter. A high resistance to both herbicides indicates a long history of use (>5 years) since resistance to propanil was first confirmed in 1989 and multiple resistance to quinclorac in 1999 [10]. The second population, ECO-S, was used as a susceptible standard for contrasting with ECO-R. ECO-S was susceptible to all rice herbicides that were tested. Purified seeds (at least 4th generation) of ECO-R were produced by treatment with 1X (0.56 kg ha^−1^) quinclorac (Facet L). The seed lot was confirmed to be 100% quinclorac-resistant and was used for RNA-seq. Seeds of both lines were germinated in pots containing potting soil (Sungro Horticulture, Agawam, MA, USA) kept in a growth chamber set to a 14 h day length and 33 °C/24 °C day/night temperature to simulate environmental conditions early in the rice growing season. For the RNA-seq experiment, a single plant was grown per pot, and each treatment had two biological replicates (Table S8). At the two-leaf stage (two leaf collars visible), 1x quinclorac + 1% *v/v* crop oil concentrate was applied at 187 L ha^−1^ in a spray chamber with a motorized boom sprayer. ECO-R and ECO-S plants were treated at the same time. After approximately 30 min, allowing for the spray droplets to dry, the treated and nontreated plants were placed back in the growth chamber. At 24 h after application, the aboveground tissues of each plant were harvested and immediately frozen in liquid nitrogen. Tissues were collected 24 h after quinclorac application because previous experiments in our laboratory showed that the majority of absorption of quinclorac occurred at least by 24HAT^.^ The samples were then transferred to RNAlater™-ICE (Invitrogen, Carlsbad, CA, USA) and shipped to the Clemson University Genomics Institute (CUGI), Clemson, South Carolina.

### 5.2. RNA Sequencing, Transcriptome Assembly, and Functional Annotation

RNA was extracted from the leaf tissues using a Total RNA kit I (Omega Bio-Tek, Inc., Norcross, GA, USA) according to the manufacturer′s instructions. The extracted RNA was treated with DNase (Invitrogen, Calsbad, CA, USA) prior to sequencing at CUGI. For library preparation, the TruSeq Stranded Total RNA kit (Illumina Inc., San Diego, CA, USA) was used according to manufacturer′s instructions to produce a paired-end library for sequencing. The samples were sequenced on an Illumina Hiseq 2500 platform. A de novo transcriptome was assembled using the Trinity RNA-Seq pipeline (Broad Institute, Cambridge, MA, USA). The raw data were assessed for quality using FastQC (Babraham Institute, Cambridge, UK) and then processed using trimmomatic to remove adapter sequences and low-quality bases using a sliding window method [69]. The processed data were then run using FastQC to verify high-quality reads. Using the TrinityRNASeq 2.2.0 software, the samples were normalized by replication using a coverage size of 100 and a kmer of 32. The normalized reads were then assembled as transcripts and genes using Trinity with the stranded library set as the default. Transdecoder 3.0.1 (https://github.com/TransDecoder/TransDecoder, accessed on 1 March 2021) as used to scan the transcriptome for one open reading frame based on homology from the blastP database and to identify existing proteins using HMM Scan against pfam; transcripts matching both criteria were retained. CD-HIT-EST (Sanforn Burnham Prebys Medical Discovery Institute, San Diego, CA, USA) was used to cluster the transcripts based on sequence identity and sequences with 98% or greater similarity were retained. The transcriptome was assessed for transcriptome completeness using BUSCO (University of Geneva, Geneva, CH). The protein sequence for each transcript was functionally annotated using the Eukaryotic Non-Model Transcriptome Annotation Pipeline (EnTAP v 0.10.8) with runP and the following databases: plant, eggnog proteins, uniport_sprot, and uniport_trembl.

### 5.3. Gene Ontology Analysis

A gene ontology enrichment analysis was performed with ENTAP GO annotations, and the list of differentially expressed genes from each comparison using the rank-based gene ontology analysis with adaptive clustering tool GO_MWU [70] in R programming language. The tool was run using the standard Fisher′s exact test mode by setting the table of interest as a binary variable to indicate whether the genes were differentially expressed or not. Enrichment analyses were performed on molecular function, biological processes, and cellular components separately for each comparison. A term was considered statistically significantly enriched if the false discovery rate and adjusted *p*-value were less than 0.05. To better characterize the resulting ontological terms and describe the interconnected pathways within the treatments, the results were analyzed and visualized using REVIGO (http://revigo.irb.hr/, accessed on 26 November 2021) and CirGO, respectively [71]. Descriptions of the gene ontology terms and the functions of the terms were obtained from the EggNOG [72] and GO Consortium [73] databases.

### 5.4. Differential Gene Expression

Samples that had trimmed reads greater than 20% over minimum were downsampled using seqtk sample (v1.3). Alignment files for each of the 8 samples were generated using RSEM (https://github.com/lh3/seqtk, accessed on 21 February 2020) (v1.3.3) with the bowtie2 and reverse strandedness options. The de novo transcriptome was used as the reference. A GTF file of the transcripts was generated as a boundary for comparing each sample to the reference transcriptome. Subread’s feature count (http://subread.sourceforge.net/, accessed on 28 March 2021) (v2.0.2) was used to count only the unique and concordant aligned reads. The Bioconductor (https://www.bioconductor.org/, accessed on 28 July 2021) package edgeR, developed for use with the R statistical software program (https://www.r-project.org/, accessed on 21 July 2021), was used to quantify the filtered raw counts produced from the RNA sequencing [74,75]. A standard normalization using trimmed mean of M-values (TMM) was applied to the counts. The counts were fit using a GLM model for the determination of significance (*p* ≤ 0.01) and a quasi-likelihood F-test for specific comparisons of interest in the experiment. The resulting analysis was then evaluated using a false discovery rate for *p*-value correction to reduce the error in the results. The multi-dimensional scaling (MDS) plot was generated to assess the disparity of the replications for each treatment and accession. Volcano plots, for visual assessment of gene expression, and a table of log2-fold changes with respective genes within the comparisons of interest were generated from the analysis. To minimize the influence of differential genomic background on differential gene expression between the resistant and susceptible lines, we compared the fold-change of differentially expressed genes within the same genotype in addition to comparison between ECO-S and ECO-R. The results were then used in the subsequent descriptive analysis to describe the patterns of expression within the tested conditions. To reduce the number of potential genes used in describing the expression patterns, categories or groupings were assigned to the sets of differentially expressed genes. The descriptions of the genes and pathways are based on the data in the Uniprot [76] and KEGG databases [77]. Three methods (gatk, freebayes, and samtools) were used to predict the SNPs present in TIR1.

### 5.5. Validation of Gene Expression by RT-qPCR

Seeds from the same ECO-R and ECO-S lines used for the RNA sequencing experiment were grown and sprayed with quinclorac in an identical manner as the RNA-seq experiment. The experimental unit was a pot containing a single plant. Each treatment was replicated three times. At the two-leaf growth stage, the treated plants were sprayed using the same quinclorac and adjuvant batch as described previously. Tissues of the nontreated and treated plants were harvested and immediately frozen in liquid nitrogen and stored at −80 °C until processed. Total RNA was isolated using an E.Z.N.A ^®^ DNA/RNA isolation kit (Omega Biotek, Norcross, GA) and was converted to cDNA using a RevertAid First Strand cDNA Synthesis Kit (Thermo Fisher Scientific, Waltham, MA, USA) according to the manufacturer’s instructions using 1 μg of total RNA for each sample. The differences in the transcript abundance of eight genes, *UGT75D1, TPP, TPS6, UGT73D1, UGT73E1, CYP709B1, CYP709B2,* and *TIR1,* were validated by RT-qPCR using the iCycler Real-Time PCR Detection System (Bio-Rad). Each qPCR reaction contained 1X IQ^TM^ SYBR Green Superpmix (2x) (BIO-RAD), 1 μL of cDNA (1:5 diluted), and 0.5 μM of gene-specific primers (Table S9). The relative expression levels of each gene were calculated using the 2^−ΔΔCt^ algorithm [78] by normalizing to the expression of the *O. sativa* elongation factor 2 gene, which was used as an internal control. Each sample was analyzed in two technical replicates using three biological replicates. The primer efficiency of *OsEF2* was calculated based on a standard curve generated using a ten-fold dilution of *Echinochloa* cDNA over four dilution points using three technical replicates, and it was found to be 101%.

## Figures and Tables

**Table 1:** Comparison of putative genes (with *p*-value < 0.01, FDR < 0.05) involved in detoxification after quinclorac treatment. The increase in expression of putative genes in quinclorac-resistant *Echinochloa colona* (ECO-R) compared to the susceptible genotype (ECO-S) 24 h after treatment.

	ECO-R			ECO-S		
Gene Family	Name	No. of Transcripts	Av. Fold Change	Name	No. of Transcripts	Av. Fold Change
Cytochrome	**CYP709B1**	9	9.5	**CYP71A8**	1	338.7
P450	**CYP709B1**	7	7.6	**unknown**	5	12.2
	**CYP72A1**	3	3.3	**CYP71A1**	2	107
	**CYP72C1**	2	10.4	**CYP71A6**	2	4.4
	**CYP72A14**	4	11.9	**CYP71A9**	1	46
	**CYP72A15**	11	7.3	**CYP89A2**	6	5.6
	**CYP94C1**	6	7.2	CYP71B3	2	DR
	**unknown**	3	8.9	CYP71C2	1	DR
	CYP71A1	7	10.6	CYP71D8	1	DR
	CYP71A6	2	5.7	CYP87A3	4	DR
	CYP71A9	3	14.7			
	**CYP71A21**	2	7.0			
	CYP71B3	3	24.1			
	CYP71C2	4	5.7			
	CYP71D8	3	7.5			
	CYP87A3	4	316.8			
	CYP94B3	5	8.5			
UDP-	U72B1	2	6.9	U74F2	7	2.6
glucoronosyl	**U73C1**	2	9.4	U72B1	3	DR
and	**U73C2**	5	3.8	U73C3	6	DR
UDP-glucosyl	U73C3	5	5.5	**U73C6**	1	4.3
transferase	**U73D1**	5	8.1	U73E1	1	10.9
	U73E1	6	3.1	U75D1	3	DR
	U74F2	2	5.0	U75D1-like	4	DR
	U75D1	3	17.0	**U76E3**	2	11.6
	U75D1-like	10	5.8	**U83A1**	8	6.1
	**U76F1**	1	4.4	**U88A1**	3	9.6
	U83A1	11	4.2	unknown	13	13.8
	**U85A2**	3	3.6			
	**U88F5**	2	2.2			
ABC	**unknown B**	3	3.2	**AB5A**	1	24
Transporters	**AB20B**	1	3.9	**AB11B**	1	8.8
	**AB22G**	1	3.5	**AB39G**	2	12.9
	**AB48G**	1	383.3	**unknown**	13	4.5
	**AB53G**	1	3.2	unknown D	7	DR
	**AB5G**	4	4.9	AB14C	2	DR
	**AB6B**	1	2.5	AB25B	7	DR
	**unknown C**	7	4.3	AB26B	2	DR
	**AB1D**	4	3.3	AB2C	1	DR
	unknown D	12	3.5	AB4C	3	DR
	AB14C	3	3.5	AB6I	6	DR
	AB25B	8	2.7	AB7A	1	DR
	AB26B	5	5.1	AB7G	2	DR
	AB2C	1	4.2	AB10I	1	DR
	AB4C	4	4.6			
	AB6I	7	2.8			
	AB7A	5	3.2			
	AB7G	2	3.6			
	AB10I	2	4.2			

DR = Downregulated. Genes in bold are unique to each genotype.

## Data Availability

The RNA-seq data generated in this study are available at BioProject PRJNA398073. The published transcriptome is available at BioProject PRJNA512635. The transcriptome data generated in this study will be deposited and publicly available upon the acceptance of the manuscript.

## Supplementary Materials

The following supporting information can be downloaded at: https://www.mdpi.com/article/10.3390/genes13030515/s1. Table S1: Summary of the results of the de novo transcriptome assembly analysis. Table S2: Summary of gene annotation for assembly of the de novo transciptome for the comparisons between ECO-R and ECO-S. Table S3: Summary of the repression or induction of genes in different fold-change categories from the differential gene expression analysis for the comparisons between ECO-R and ECO-S. Table S4: Summary of significantly upregulated genes in nontreated ECO-R compared to nontreated ECO-S (*p* value < 0.01, FDR < 0.05). Table S5: Result of GO enrichment analysis using quinclorac treated ECO-S transcriptome. Table S6: Comparison of ethylene signaling genes between ECO-R and ECO-S after quinclorac treatment (*p* value < 0.01, FDR < 0.05). Table S7: List of genes upregulated in ECO-R after quinclorac treatment (*p* value < 0.01, FDR < 0.05). Table S8: Treatment structure for for the RNA-seq experiment. Table S9: Primers used for the qRT-PCR gene expression assay with and without quinclorac treatment. Figure S1: Diagram depicting the quinclorac-activated physiological pathway as explained in the literature [21] and the response of ECO-S and ECO-R, 24 h after treatment, as deduced by the transcriptome sequence analysis.
