# Supplementary material for: High Resistance to Quinclorac in Multiple-Resistant Echinochloa colona Associated with Elevated Stress Tolerance Gene Expression and Enriched Xenobiotic Detoxification Pathway
Source: Genes (Basel). 2022 Mar 15;13(3):515. doi: 10.3390/genes13030515 (PMC8949966; doi:10.3390/genes13030515)
Supplement: Supplementary file 1 [file genes-13-00515-s001.zip › genes-1585357-supplementary.pdf]

High resistance to quinclorac in multiple-resistant *Echinochloa colona* associated with elevated stress tolerance gene expression and enriched xenobiotic detoxification pathway

Gulab Rangani, Christopher E. Rouse, Christopher Saski, Rooksana E. Noorai, Vijay Shankar, Amy L. Lawton- Rauh, Isabel S. Werle, Nilda Roma-Burgos

Supplemental Tables

Table S1: Summary of the results of the *de novo* transcriptome assembly analysis.

Transcriptome Component	Size/ Length
Illumina raw read pairs	544,870,782
Number of Transcripts	97,968
Annotated sequences (blastX)	62,192
Assembled genes	49,425
Number of bases	250
Read Length (n)	125

Table S2: Summary of gene annotation for assembly of the *de novo* transciptome for the comparisons between ECO-R and ECO-S.

Annotation	Expression	Nontreated ECO-R vs Nontreated ECO-S	Treated ECO-R vs Nontreated ECO-R	Treated ECO-S vs Nontreated ECO-S	Treated ECO-R vs Treated ECO-S
Annotated	Up-regulated	9,056	11,779	5,742	8,575
	Down-regulated	10,247	10,620	4,758	10,448
	Total	19,303	22,399	10,500	19,023
Non-Annotated	Up-regulated	168	318	161	229
	Down-regulated	7	0	81	219
	Total	175	318	242	448
Total	Up-regulated	9,224	12,097	5,903	8,804
	Down-regulated	10,254	10,620	4,839	10,667
	Total	19478	22,717	10,742	19471

Table S3: Summary of the repression or induction of genes in different fold-change categories from the differential gene expression analysis for the comparisons between ECO-R and ECO-S.

Fold Change Category (log FC)	Nontreated ECO-R vs Nontreated ECO-S		Treated ECO-R vs Nontreated ECO-R		Treated ECO-S vs Nontreated ECO-S		Treated ECO-R vs treated ECO-S	
	Up-regulated	Down-regulated	Up-regulated	Down-regulated	Up-regulated	Down-regulated	Up-regulated	Down-regulated
1-2	4,033	4,249	4,447	4,542	2,306	2,289	5,610	5,539
3-4	1,322	1,354	1,882	1,603	1,020	588	520	1,139
5-6	120	219	403	211	205	51	46	293
7-8	30	170	205	38	59	20	21	130
9-10	24	57	108	18	12	2	8	50
≥11	5	20	44	0	2	1	2	13

Table S4: Summary of significantly upregulated genes in nontreated ECO-R compared to nontreated ECO-S (pvalue < 0.01, FDR < 0.05).

Biological process	Name of genes	No. of Transcripts	Av. log FC
**Photosynthesis**	PsaA and PsaB bind P700, the primary electron donor of photosystem I (PSI)	2	8
	ATP synthase subunits	2	7.4
	Cytochrome b561	2	3.4
	Photosystem II CP47 reaction center protein	2	8.4
**Nitrogen assimilation**	Nitrate reductase	5	2.6
	aspartate aminotransferase	6	2
	asparagine synthetase	5	3.3
	alanine aminotransferase	2	2.0
	transketolase 1		5.4
**Trehalose biosynthesis**	Alpha, alpha-TPS [UDP-forming] 5	1	4
	Alpha, alpha-TPS [UDP-forming] 1	3	2.5
	Probable alpha,alpha- TPS [UDP-forming] 7	1	2.7
	Probable alpha,alpha- TPS [UDP-forming] (unknown)	2	2.4
	Probable alpha,alpha- TPS [UDP-forming] 10	1	3.5
**Ureide Metabolism**	xanthine dehydrogenase	2	3.5
	Catalyzes the oxidation of uric acid to 5hydroxyisourate	4	2.2
	5-hydroxyisourate hydrolase	3	1.6
	Probable ureidoglycolate hydrolase	4	1.7
	Probable (S)-ureidoglycine aminohydrolase	4	2
**Ethylene biosynthesis**	ACC-oxidase	11	2.7
	elevated ethylene insensitive protein (EIN) 3	3	2.2
	ERF1	2	2.6
	ERF73	1	2
**Plant abiotic stress signaling**	ABA receptor protein PYL8	2	1.5
	CPK5	2	2.2
	ALPL1-like	3	2.3
**Cytochrome P450**	CYP unknown	14	3.2
	CYP704C1	6	2.6
	CYP71A3	4	2.3
	CYP76AD1	1	3.6
	CYP76B6	1	4.4
	CYP76C1	2	3.2
	CYP78A6	6	4.1
	81F1	3	3.4
	81F4	6	2.9
	89A2	7	1.8
**UDP-Glycosyltransferase**	Cyanidin 3-O-rutinoside 5-O-glucosyltransferase	7	4.2
	75D1-like	7	2.6
	Unknown	9	3.2
	U71K2	3	2.0
	U73C1	3	2.2
	U73D1	3	3.7
	U74F2	7	3.5
	U89B1	3	4.8
	Crocetin glucosyltransferase	3	5.3
**ABC Transporters**	AB10C	5	3.7
	AB14C	5	4.7

	AB48G	1	5.7
**Biological process**	**Name of genes**	**No. of Transcripts**	**Av. log FC**
	AB53G	2	4.1
	AB22G	3	3
	AB36G	3	3.5
	AB24G	4	2.9
	AB2C	3	3
	AB3C	2	3
	AB3B	5	3.2

Table S5: Result of GO enrichment analysis using quinclorac treated ECO-S transcriptome

Type	pValue	DEGs	nseqs in the category (ref)	Enriched term	Name
Biological Processes	1.28E-46	**237**	699	GO:0015979	photosynthesis
	1.38E-31	**842**	4585	GO:0010035	response to inorganic substance
	2.90E-31	**125**	317	GO:0019684	photosynthesis, light reaction
	8.15E-31	**423**	1917	GO:0006091	generation of precursor metabolites and energy
	2.78E-26	**525**	2652	GO:0098542	defense response to other organism
	6.46E-23	**557**	2950	GO:0010038;GO:0046686	response to metal ion
	2.23E-22	**72**	157	GO:0009767	photosynthetic electron transport chain
	1.85E-21	**50**	85	GO:0019253;GO:0019685	photosynthesis, dark reaction
	9.58E-21	**58**	114	GO:0015977	carbon fixation
	1.10E-19	**794**	4676	GO:0009607;GO:0051707;GO:0043207;GO:0044419	biological process involved in interspecies interaction between organisms
	5.85E-19	**94**	271	GO:0016108;GO:0016109;GO:0016116;GO:0016117	carotenoid metabolic process
	3.40E-18	**374**	1895	GO:0009617;GO:0042742	response to bacterium
	1.24E-17	**92**	273	GO:0005982	starch metabolic process
	9.08E-17	**106**	348	GO:0043094	cellular metabolic compound salvage
	5.78E-16	**179**	751	GO:0022900	electron transport chain
	1.09E-15	**40**	73	GO:0009773	photosynthetic electron transport in
	4.34E-14	**24**	31	GO:0030505	inorganic diphosphate transport
	2.47E-13	**161**	692	GO:0009657	plastid organization
	1.19E-12	**77**	247	GO:0000097	sulfur amino acid biosynthetic process
	2.06E-12	**53**	139	GO:0009853	photorespiration
	4.48E-12	**849**	5424	GO:0006082;GO:0019752;GO:0043436	organic acid metabolic process
	1.14E-11	**92**	334	GO:0009873	ethylene-activated signaling pathway
	1.56E-11	**868**	5594	GO:1901700	response to oxygen-containing compound
	1.96E-11	**24**	37	GO:0015714;GO:0015717;GO:0035436	triose phosphate transmembrane transport
	2.18E-11	**883**	5714	GO:0009605	response to external stimulus
	6.62E-11	**394**	2255	GO:0006979	response to oxidative stress
	2.40E-10	**47**	128	GO:0019252	starch biosynthetic process
	3.80E-10	**388**	2243	GO:0009266	response to temperature stimulus
	1.35E-09	**87**	335	GO:0000096	sulfur amino acid metabolic process
	1.45E-09	**33**	75	GO:0019509;GO:0071267;GO:0043102;GO:0071265	L-methionine biosynthetic process
	5.44E-09	**757**	4933	GO:0006952	defense response
	5.76E-09	**762**	4972	GO:0009719;GO:0009725	response to endogenous stimulus
	1.06E-08	**34**	84	GO:0016119	carotene metabolic process
	1.31E-08	**106**	458	GO:0071369	cellular response to ethylene stimulus
	1.31E-08	**156**	760	GO:0008299;GO:0016114	isoprenoid biosynthetic process
	1.56E-08	**146**	700	GO:0006006;GO:0006007;GO:0019320;GO:0046365	glucose metabolic process
	1.56E-08	**67**	243	GO:0006081	cellular aldehyde metabolic process
	2.85E-08	**176**	895	GO:0050832	defense response to fungus
	3.52E-08	**80**	318	GO:0003333;GO:1905039;GO:1903825	carboxylic acid transmembrane transport
	4.38E-08	**187**	971	GO:0006720;GO:0006721	isoprenoid metabolic process
	4.38E-08	**116**	528	GO:0042542	response to hydrogen peroxide
	8.15E-08	**510**	3211	GO:0033993	response to lipid
	1.10E-07	**11**	12	GO:0048838;GO:0097438	release of seed from dormancy
	1.69E-07	**216**	1179	GO:0016051	carbohydrate biosynthetic process
	1.90E-07	**16**	25	GO:0015713;GO:0042873	phosphoglycerate transmembrane transport

	1.97E-07	**125**	597	GO:0009135;GO:0009132;GO:0009179	pyruvate metabolic process
	2.06E-07	**907**	6161	GO:0010033	response to organic substance
	2.07E-07	**26**	60	GO:0009854	oxidative photosynthetic carbon pathway
	2.21E-07	**45**	145	GO:0015995	chlorophyll biosynthetic process
	2.22E-07	**433**	2686	GO:0009314;GO:0009416	response to radiation
	2.89E-07	**104**	474	GO:0009658	chloroplast organization
	2.89E-07	**25**	57	GO:0019288;GO:0019682	glyceraldehyde-3-phosphate metabolic process
	3.14E-07	**90**	392	GO:0006778;GO:0033013	tetrapyrrole metabolic process
	3.37E-07	**276**	1597	GO:1901605	alpha-amino acid metabolic process
	3.58E-07	**16**	26	GO:0006522;GO:0009078	pyruvate family amino acid metabolic process
	3.72E-07	**265**	1524	GO:0051186	obsolete cofactor metabolic process
	3.97E-07	**228**	1275	GO:0009620	response to fungus
	3.97E-07	**221**	1228	GO:0016052	carbohydrate catabolic process
	4.07E-07	**63**	242	GO:0015994	chlorophyll metabolic process
	4.28E-07	**35**	101	GO:0006094	gluconeogenesis
	5.34E-07	**245**	1395	GO:0006955;GO:0045087	immune response
	5.41E-07	**69**	277	GO:0006779;GO:0033014	tetrapyrrole biosynthetic process
	5.91E-07	**92**	410	GO:0009642	response to light intensity
	7.13E-07	**159**	829	GO:0006073;GO:0044042	cellular glucan metabolic process
	7.13E-07	**28**	72	GO:0010189;GO:0042360	vitamin E metabolic process
	7.63E-07	**337**	2038	GO:0044262	cellular carbohydrate metabolic process
	1.05E-06	**196**	1077	GO:0005996;GO:0019318	monosaccharide metabolic process
	1.11E-06	**603**	3963	GO:0044283	small molecule biosynthetic process
	1.19E-06	**155**	810	GO:0009723	response to ethylene
	1.33E-06	**166**	883	GO:0046148;GO:0042440	pigment metabolic process
	1.38E-06	**14**	22	GO:0019255	glucose 1-phosphate metabolic process
	1.38E-06	**14**	22	GO:0019388	galactose catabolic process
	1.38E-06	**14**	22	GO:0035999	tetrahydrofolate interconversion
	1.44E-06	**257**	1496	GO:0009409	response to cold
	1.57E-06	**83**	366	GO:0006865	amino acid transport
	1.69E-06	**116**	567	GO:0000160	phosphorelay signal transduction system
	1.71E-06	**39**	126	GO:0009668;GO:0010027	plastid membrane organization
	2.08E-06	**31**	89	GO:0009765	photosynthesis, light harvesting
	2.35E-06	**12**	17	GO:0006524;GO:0009080;GO:0042853;GO:0042851	alanine catabolic process
	2.63E-06	**167**	900	GO:0000302	response to reactive oxygen species
	3.20E-06	**9**	10	GO:0015729	oxaloacetate transport
	3.67E-06	**29**	82	GO:0009240;GO:0046490	isopentenyl diphosphate biosynthetic process
	3.91E-06	**18**	37	GO:0010207	photosystem II assembly
	4.30E-06	**454**	2916	GO:0006970;GO:0009651	response to osmotic stress
	5.50E-06	**59**	238	GO:0009644	response to high light intensity
	5.62E-06	**14**	24	GO:0010275	NAD(P)H dehydrogenase complex assembly
	6.22E-06	**143**	755	GO:0009753;GO:0070542	response to fatty acid
	8.36E-06	**29**	85	GO:0016122	xanthophyll metabolic process
	1.15E-05	**44**	161	GO:0009627	systemic acquired resistance
	1.18E-05	**11**	16	GO:0030912	response to deep water
	1.22E-05	**61**	255	GO:0010118	stomatal movement
	1.22E-05	**71**	313	GO:0009069	serine family amino acid metabolic process
	1.26E-05	**28**	82	GO:0006108	malate metabolic process
	1.63E-05	**211**	1225	GO:0008652;GO:1901607	cellular amino acid biosynthetic process
	1.79E-05	**29**	88	GO:0019344	cysteine biosynthetic process
	1.89E-05	**762**	5244	GO:0005975	carbohydrate metabolic process
	1.89E-05	**40**	143	GO:0045036;GO:0072596;GO:0072598	protein localization to chloroplast
	1.89E-05	**100**	494	GO:0043648	dicarboxylic acid metabolic process
	1.91E-05	**8**	9	GO:0009915	phloem sucrose loading
	1.91E-05	**7**	7	GO:0051973	positive regulation of telomerase activity
	2.19E-05	**235**	1397	GO:0006820	anion transport
	2.19E-05	**10**	14	GO:0030388	fructose 1,6-bisphosphate metabolic process

	2.24E-05	**19**	45	GO:0032544	plastid translation
	2.58E-05	**11**	17	GO:0010036;GO:0080029;GO:0080169	response to boron-containing substance
	2.84E-05	**57**	239	GO:0042743;GO:0042744	hydrogen peroxide metabolic process
	3.24E-05	**14**	27	GO:0080005	photosystem stoichiometry adjustment
	3.32E-05	**28**	86	GO:0033559;GO:0006636	unsaturated fatty acid metabolic process
	3.35E-05	**38**	136	GO:0006563	L-serine metabolic process
	3.49E-05	**146**	800	GO:0009639	response to red or far red light
	3.64E-05	**797**	5536	GO:0055085	transmembrane transport
	5.06E-05	**49**	198	GO:0006555;GO:0009086	methionine metabolic process
	5.32E-05	**14**	28	GO:0010117	photoprotection
	5.32E-05	**10**	15	GO:0010236	plastoquinone biosynthetic process
	5.32E-05	**11**	18	GO:0060919	auxin influx
	5.32E-05	**14**	28	GO:0071836	nectar secretion
	5.83E-05	**62**	274	GO:0098656	anion transmembrane transport
	5.93E-05	**20**	52	GO:0016120	carotene biosynthetic process
	6.03E-05	**152**	849	GO:0009408	response to heat
	6.03E-05	**385**	2490	GO:0097305;GO:0009737	response to alcohol
	6.84E-05	**390**	2530	GO:0032787	monocarboxylic acid metabolic process
	7.83E-05	**34**	120	GO:0042214	terpene metabolic process
	9.68E-05	**15**	33	GO:0009590	detection of gravity
	0.00013697	**40**	155	GO:0009070	serine family amino acid biosynthetic process
	0.00013697	**55**	240	GO:0010114	response to red light
	0.0001387	**51**	217	GO:0006099	tricarboxylic acid cycle
	0.0001387	**71**	336	GO:0010200	response to chitin
	0.0001708	**54**	236	GO:0046364	monosaccharide biosynthetic process
	0.00019447	**13**	27	GO:0019284;GO:0033353	S-adenosylmethionine cycle
	0.00020913	**152**	869	GO:0080167	response to karrikin
	0.000215	**14**	31	GO:0070206	protein trimerization
	0.00022144	**15**	35	GO:0016139	glycoside catabolic process
	0.00026137	**18**	48	GO:0009435	NAD biosynthetic process
	0.00026137	**112**	605	GO:0009624	response to nematode
	0.00027695	**34**	127	GO:0006534	cysteine metabolic process
	0.00030514	**13**	28	GO:0009871	jasmonic acid and ethylene-dependent systemic resistance, ethylene mediated signaling pathway
	0.00031363	**209**	1271	GO:0005976;GO:0044264	polysaccharide metabolic process
	0.00034594	**11**	21	GO:0019676;GO:0019740	ammonia assimilation cycle
	0.00039682	**7**	9	GO:0080158	obsolete chloroplast ribulose bisphosphate carboxylase complex biogenesis
	0.00040336	**30**	108	GO:0006775;GO:0042362	fat-soluble vitamin metabolic process
	0.00040462	**49**	214	GO:0009637	response to blue light
	0.00044198	**117**	646	GO:0008643	carbohydrate transport
	0.00050791	**9**	15	GO:0052863;GO:0052865	process
	0.00051381	**8**	12	GO:2000037	regulation of stomatal complex patterning
	0.00058449	**11**	22	GO:0110126	phloem loading
	0.00066813	**6**	7	GO:0034051	negative regulation of plant-type hypersensitive
	0.00071047	**60**	285	GO:0006814	sodium ion transport
	0.00085249	**5**	5	GO:0019379;GO:0019419	sulfate reduction
	0.00090956	**79**	407	GO:0006733;GO:0019362;GO:0072524;GO:0046496	obsolete oxidoreduction coenzyme metabolic process
	0.0009329	**563**	3900	GO:0006811;GO:0034220	ion transport
	0.00095507	**11**	23	GO:0044375	regulation of peroxisome size
	0.00099895	**14**	35	GO:0009864	induced systemic resistance, jasmonic acid
	0.00103273	**12**	27	GO:0045824	negative regulation of innate immune response
	0.00110276	**217**	1356	GO:0009414;GO:0009415;GO:0001101	response to acid chemical
	0.00111316	**412**	2778	GO:0006520	cellular amino acid metabolic process
	0.00120915	**35**	142	GO:0010109	regulation of photosynthesis
	0.00125281	**24**	83	GO:0015766;GO:0015772;GO:0015770	oligosaccharide transport

	0.00126348	**36**	148	GO:0019319	hexose biosynthetic process
	0.00142323	**50**	231	GO:0015718	monocarboxylic acid transport
	0.00148057	**16**	45	GO:0080024;GO:0018874	indolebutyric acid metabolic process
	0.00167064	**17**	50	GO:0000105	histidine biosynthetic process
	0.00177579	**45**	203	GO:0010218	response to far red light
	0.00189494	**161**	973	GO:0015711;GO:0046942;GO:0015849	organic anion transport
	0.00193208	**22**	75	GO:0046501;GO:0006782	protoporphyrinogen IX metabolic process
	0.00210521	**10**	21	GO:0070207	protein homotrimerization
	0.00212613	**57**	278	GO:0009067	aspartate family amino acid biosynthetic process
	0.00222397	**12**	29	GO:0120256;GO:0016107;GO:0043290;GO:0046345	sesquiterpenoid catabolic process
	0.00263061	**149**	896	GO:0044272	sulfur compound biosynthetic process
	0.0026357	**283**	1856	GO:0002376	immune system process
	0.0026357	**24**	87	GO:0010167	response to nitrate
	0.00287207	**18**	57	GO:0042939	tripeptide transport
	0.00287207	**18**	57	GO:0046037;GO:0006177	GMP metabolic process
	0.00292508	**19**	62	GO:0006535	cysteine biosynthetic process from serine
	0.00326441	**113**	652	GO:0046939	nucleotide phosphorylation
	0.00343374	**22**	78	GO:0046653	tetrahydrofolate metabolic process
	0.00360237	**18**	58	GO:0090332	stomatal closure
	0.00361252	**72**	380	GO:0009066	aspartate family amino acid metabolic process
	0.00381076	**5**	6	GO:0052889	9,9'-di-cis-zeta-carotene desaturation to 7,9,7',9'-
	0.00409353	**22**	79	GO:0009861	jasmonic acid and ethylene-dependent systemic
	0.00409353	**78**	421	GO:0010150;GO:0090693;GO:0010260	animal organ senescence
	0.00426855	**537**	3769	GO:0071310;GO:0070887	cellular response to chemical stimulus
	0.00435557	**20**	69	GO:0046482	para-aminobenzoic acid metabolic process
	0.00456409	**9**	19	GO:0042425	choline biosynthetic process
	0.00456409	**9**	19	GO:0090322	regulation of superoxide metabolic process
	0.00458161	**385**	2629	GO:0046394;GO:0016053	organic acid biosynthetic process
	0.00479555	**345**	2333	GO:0032870;GO:0071495;GO:0009755	cellular response to endogenous stimulus
	0.00489845	**166**	1030	GO:0051188	obsolete cofactor biosynthetic process
	0.00497471	**6**	9	GO:0080093	regulation of photorespiration
	0.00499312	**21**	75	GO:0006097;GO:0046487	glyoxylate metabolic process
	0.005557	**30**	125	GO:0006544	glycine metabolic process
	0.00598286	**21**	76	GO:0009635	response to herbicide
	0.00598286	**59**	303	GO:0031348	negative regulation of defense response
	0.00598286	**21**	76	GO:0043090	amino acid import
	0.00623981	**48**	234	GO:0002237	response to molecule of bacterial origin
	0.00645814	**11**	28	GO:0000103	sulfate assimilation
	0.00645814	**11**	28	GO:0071423	malate transmembrane transport
	0.00800424	**29**	122	GO:0072525	pyridine-containing compound biosynthetic
	0.00822271	**17**	57	GO:0010588	cotyledon vascular tissue pattern formation
	0.00833011	**7**	13	GO:0019605	butyrate metabolic process
	0.00833011	**7**	13	GO:0032928	regulation of superoxide anion generation
	0.00882395	**57**	295	GO:0016998	cell wall macromolecule catabolic process
	0.00897175	**11**	29	GO:0034635	glutathione transport
	0.00917349	**58**	302	GO:0070301	cellular response to hydrogen peroxide
	0.01000068	**17**	58	GO:0010187	negative regulation of seed germination
	0.01020266	**6**	10	GO:0031221;GO:0031222;GO:2000895	arabinan metabolic process
	0.01027974	**9**	21	GO:0019357;GO:0046497;GO:0019358;GO:0019365	pyridine nucleotide salvage
	0.01094975	**79**	443	GO:0009060;GO:0045333	cellular respiration
	0.01094975	**43**	209	GO:0033037;GO:0052386;GO:0052482;GO:0052542;GO:0052545;GO:0052543;GO:0052544	polysaccharide localization

	0.01151186	**13**	39	GO:0048829	root cap development
	0.01192083	**47**	235	GO:0120252;GO:0120251	hydrocarbon metabolic process
	0.01227435	**172**	1096	GO:0048366	leaf development
	0.01313803	**38**	180	GO:0009867;GO:0071395	cellular response to jasmonic acid stimulus
	0.01365602	**23**	92	GO:0015985;GO:0015986	energy coupled proton transport, down
	0.01420113	**67**	367	GO:0015748	organophosphate ester transport
	0.01588768	**19**	71	GO:0002832	negative regulation of response to biotic
	0.01743419	**22**	88	GO:0009682	induced systemic resistance
	0.01760791	**175**	1127	GO:0006732	obsolete coenzyme metabolic process
	0.01787938	**12**	36	GO:0010232;GO:0010233	vascular transport
	0.01838185	**13**	41	GO:0001906;GO:0031640;GO:0044364	cell killing
	0.01838185	**6**	11	GO:0009438;GO:0051596	methylglyoxal metabolic process
	0.01838185	**6**	11	GO:0009823	cytokinin catabolic process
	0.01838185	**6**	11	GO:0010113	negative regulation of systemic acquired
	0.01838185	**6**	11	GO:0019481	L-alanine catabolic process, by transamination
	0.01838185	**74**	418	GO:0071482;GO:0071478	cellular response to radiation
	0.01962771	**18**	67	GO:0006547;GO:0052803	histidine metabolic process
	0.02013626	**4**	5	GO:0009439;GO:0009440	cyanate catabolic process
	0.02013626	**4**	5	GO:0044046;GO:0052047;GO:0052048;GO:0052051;GO:0052211	obsolete interaction with host via substance released outside of symbiont
	0.02013626	**4**	5	GO:1901999;GO:1902000	homogentisate catabolic process
	0.02055523	**17**	62	GO:0030149;GO:0046466;GO:0006677;GO:0019377;GO:0046477;GO:0046479;GO:0046514	membrane lipid catabolic process
	0.02119504	**24**	101	GO:0005983	starch catabolic process
	0.0212924	**19**	73	GO:0006026;GO:0006032;GO:1901072;GO:0046348	aminoglycan catabolic process
	0.02199277	**12**	37	GO:0009641	shade avoidance
	0.02199277	**12**	37	GO:0046459	short-chain fatty acid metabolic process
	0.02258733	**32**	149	GO:0009251;GO:0044247	cellular polysaccharide catabolic process
	0.0242989	**21**	85	GO:0006672	ceramide metabolic process
	0.0242989	**21**	85	GO:0006829	zinc ion transport
	0.02434006	**34**	162	GO:0042537	benzene-containing compound metabolic
	0.02660275	**11**	33	GO:0010021;GO:2000896	amylopectin biosynthetic process
	0.02720757	**24**	103	GO:1901070	guanosine-containing compound biosynthetic
	0.02804766	**13**	43	GO:0006020	inositol metabolic process
	0.02827857	**27**	121	GO:0006783;GO:0042168	heme metabolic process
	0.02849326	**17**	64	GO:0006687	glycosphingolipid metabolic process
	0.02953815	**41**	208	GO:0005985	sucrose metabolic process
	0.0323066	**7**	16	GO:0006983	ER overload response
	0.0323066	**7**	16	GO:0015803	branched-chain amino acid transport
	0.03288539	**22**	93	GO:0009696	salicylic acid metabolic process
	0.03288539	**109**	672	GO:0045454	cell redox homeostasis
	0.03288539	**255**	1736	GO:1901615	organic hydroxy compound metabolic process
	0.0330688	**14**	49	GO:0010190	cytochrome b6f complex assembly
	0.03310648	**17**	65	GO:0006777;GO:0019720;GO:0043545;GO:0051189;GO:0032324	Mo-molybdopterin cofactor metabolic process
	0.03316085	**11**	34	GO:0071323	cellular response to chitin
	0.03339188	**287**	1976	GO:1901701	cellular response to oxygen-containing
	0.03345491	**75**	436	GO:0015698	inorganic anion transport
	0.03521325	**16**	60	GO:0015706	nitrate transport
	0.03757846	**15**	55	GO:0006549;GO:0009097	isoleucine metabolic process
	0.03970345	**20**	83	GO:0002229;GO:0002239	response to oomycetes
	0.03987867	**5**	9	GO:0009828	plant-type cell wall loosening
	0.03987867	**5**	9	GO:0010201	response to continuous far red light stimulus by the high-irradiance response system

	0.04137266	**8**	21	GO:0006021	inositol biosynthetic process
	0.04137266	**11**	35	GO:0009099	valine biosynthetic process
	0.04137266	**8**	21	GO:0016110;GO:0016118;GO:0016121;GO:0046247;GO:0120253;GO:0016124	xanthophyll catabolic process
	0.04137266	**42**	219	GO:0046164;GO:1901616	organic hydroxy compound catabolic process
	0.04137266	**22**	95	GO:0090056;GO:1901401;GO:0010380;GO:1901463	regulation of tetrapyrrole metabolic process
	0.04580834	**74**	435	GO:0009626;GO:0034050;GO:0051702	biological process involved in interaction with symbiont
	0.04626313	**6**	13	GO:0070525	process
	0.0466558	**4**	6	GO:2000011	regulation of adaxial/abaxial pattern formation
	0.04683086	**18**	73	GO:0009862	mediated signaling pathway
	0.04683086	**18**	73	GO:0097164	ammonium ion metabolic process
Molecular Function	2.00E-14	**258**	1215	GO:0015291	activity
	2.38E-13	**24**	31	GO:0030504	transporter activity
	1.42E-12	**42**	90	GO:0005315	activity
	5.06E-10	**18**	23	GO:0015315;GO:0015121;GO:0089721	organophosphate:inorganic phosphate antiporter activity
	6.83E-10	**214**	1064	GO:0008509	anion transmembrane transporter activity
	1.71E-09	**457**	2686	GO:0022804	active transmembrane transporter activity
	2.63E-08	**119**	525	GO:0046943;GO:0005342	organic acid transmembrane transporter activity
	2.87E-08	**11**	11	GO:0008974	phosphoribulokinase activity
	4.50E-08	**190**	966	GO:0016209;GO:0004601;GO:0016684	antioxidant activity
	4.86E-08	**23**	44	GO:0030060	L-malate dehydrogenase activity
	1.15E-07	**36**	97	GO:0008187;GO:0008266	poly-pyrimidine tract binding
	2.14E-07	**136**	650	GO:0008514	activity
	2.14E-07	**30**	74	GO:0015301;GO:0140323	anion:anion antiporter activity
	3.13E-07	**16**	25	GO:0015120;GO:0042879	activity
	3.13E-07	**63**	234	GO:0015171	amino acid transmembrane transporter activity
	3.13E-07	**18**	31	GO:0071917	activity
	4.64E-07	**14**	20	GO:0004514;GO:0004516	(carboxylating) activity
	4.64E-07	**14**	20	GO:0004614	phosphoglucomutase activity
	5.43E-07	**15**	23	GO:0010309	activity
	5.52E-07	**20**	39	GO:0010277	chlorophyllide a oxygenase [overall] activity
	6.91E-07	**9**	9	GO:0010354	homogentisate prenyltransferase activity
	7.41E-07	**121**	576	GO:0015293	symporter activity
	7.73E-07	**10**	11	GO:0009670	triose-phosphate:phosphate antiporter activity
	9.71E-07	**15**	24	GO:0004021;GO:0047635	activity
	9.71E-07	**15**	24	GO:0033743	activity
	1.77E-06	**40**	128	GO:0050897	cobalt ion binding
	3.57E-06	**12**	17	GO:0004655	porphobilinogen synthase activity
	3.98E-06	**8**	8	GO:0008465	glycerate dehydrogenase activity
	3.98E-06	**8**	8	GO:0010471;GO:0010472;GO:0010473;GO:0010474;GO:0010475;GO:0080048	galactose-1-phosphate guanylyltransferase (GDP) activity
	4.29E-06	**9**	10	GO:0015131	oxaloacetate transmembrane transporter activity
	6.19E-06	**11**	15	GO:0010295	(+)-abscisic acid 8'-hydroxylase activity
	6.87E-06	**30**	87	GO:0016615	malate dehydrogenase activity
	7.08E-06	**41**	140	GO:0008028	transporter activity
	7.52E-06	**12**	18	GO:0046537	phosphoglycerate mutase activity
	1.19E-05	**496**	3189	GO:0015318;GO:0015075	ion transmembrane transporter activity
	1.51E-05	**11**	16	GO:0016984	ribulose-bisphosphate carboxylase activity
	2.09E-05	**15**	29	GO:0016618	hydroxypyruvate reductase activity
	2.26E-05	**20**	48	GO:0042132	activity
	2.74E-05	**10**	14	GO:0008843;GO:0016231	endochitinase activity

	2.74E-05	**113**	570	GO:0015297	antiporter activity
	2.74E-05	**35**	117	GO:0050308	sugar-phosphatase activity
	3.10E-05	**11**	17	GO:0004419	hydroxymethylglutaryl-CoA lyase activity
	3.10E-05	**15**	30	GO:0015038	glutathione disulfide oxidoreductase activity
	3.10E-05	**12**	20	GO:0046554	malate dehydrogenase (NADP+) activity
	4.28E-05	**19**	46	GO:0004337	geranyltranstransferase activity
	4.28E-05	**123**	640	GO:0016701;GO:0016702	oxidoreductase activity, acting on single donors with incorporation of molecular oxygen
	5.30E-05	**56**	234	GO:0004659	prenyltransferase activity
	5.57E-05	**16**	35	GO:0010294	abscisic acid glucosyltransferase activity
	5.57E-05	**20**	51	GO:0015355	transmembrane transporter activity
	5.75E-05	**10**	15	GO:0008905;GO:0008928	mannose-phosphate guanylyltransferase activity
	5.75E-05	**10**	15	GO:0010328	auxin influx transmembrane transporter activity
	5.75E-05	**10**	15	GO:0030527	structural constituent of chromatin
	6.05E-05	**14**	28	GO:0031219	levanase activity
	7.80E-05	**19**	48	GO:0004611	phosphoenolpyruvate carboxykinase activity
	0.00011872	**7**	8	GO:0047958	glycine:2-oxoglutarate aminotransferase activity
	0.00012792	**6**	6	GO:0010176	homogentisate phytyltransferase activity
	0.00012792	**6**	6	GO:0048529	magnesium-protoporphyrin IX monomethyl ester (oxidative) cyclase activity
	0.00016205	**18**	46	GO:0003922	GMP synthase (glutamine-hydrolyzing) activity
	0.00021781	**11**	20	GO:0004612	activity
	0.0002501	**27**	89	GO:0008061	chitin binding
	0.00039187	**11**	21	GO:0008886	glyceraldehyde-3-phosphate dehydrogenase (NADP+) (non-phosphorylating) activity
	0.0004203	**7**	9	GO:0000293;GO:0016723	ferric-chelate reductase activity
	0.0004203	**18**	49	GO:0004124	cysteine synthase activity
	0.00053345	**13**	29	GO:0004365;GO:0043891	glyceraldehyde-3-phosphate dehydrogenase (NAD(P)+) (phosphorylating) activity
	0.00054469	**9**	15	GO:0004370	glycerol kinase activity
	0.00064171	**11**	22	GO:0050347;GO:0052923	all-trans-nonaprenyl-diphosphate synthase (geranyl-diphosphate specific) activity
	0.00067968	**6**	7	GO:0050278	sedoheptulose-bisphosphatase activity
	0.00070077	**20**	60	GO:0016703	with incorporation of molecular oxygen, incorporation of one atom of oxygen (internal monooxygenases or internal mixed function
	0.00076979	**14**	34	GO:0003973	(S)-2-hydroxy-acid oxidase activity
	0.00081246	**19**	56	GO:0080002	acylglucosyltransferase activity
	0.00084603	**30**	111	GO:0050253;GO:0080030;GO:0080031	retinyl-palmitate esterase activity
	0.00098525	**11**	23	GO:0003883	CTP synthase activity
	0.00098525	**119**	662	GO:0016667	group of donors
	0.00104909	**7**	10	GO:0050518	cytidylyltransferase activity
	0.00109647	**8**	13	GO:0008670	2,4-dienoyl-CoA reductase (NADPH) activity
	0.00156971	**96**	518	GO:0015103	activity
	0.00192904	**32**	127	GO:0016884	as amido-N-donor
	0.00220877	**20**	65	GO:0016165	linoleate 13S-lipoxygenase activity
	0.00378667	**16**	48	GO:0016744	transketolase or transaldolase activity
	0.00384341	**5**	6	GO:0004604;GO:0009973	(thioredoxin) activity
	0.00384341	**5**	6	GO:0016719;GO:0052886;GO:0052887	carotene 7,8-desaturase activity
	0.00396496	**8**	15	GO:0080046	quercetin 4'-O-glucosyltransferase activity
	0.00478251	**7**	12	GO:0004373	glycogen (starch) synthase activity
	0.00478251	**9**	19	GO:0008878	activity
	0.00478251	**12**	31	GO:0009011	starch synthase activity
	0.00489189	**6**	9	GO:0004399	histidinol dehydrogenase activity
	0.00489189	**6**	9	GO:0004424	activity

	0.00489189	**6**	9	GO:0004458;GO:0019154	D-lactate dehydrogenase (cytochrome) activity
	0.00489189	**11**	27	GO:0016767	geranylgeranyltransferase activity
	0.00549267	**13**	36	GO:0052638;GO:0052640;GO:0052641	benzoic acid glucosyltransferase activity
	0.00686144	**82**	449	GO:0015036;GO:0015035	disulfide oxidoreductase activity
	0.00705726	**9**	20	GO:0000234	activity
	0.00705726	**9**	20	GO:0008661	activity
	0.00716704	**23**	87	GO:0015114	activity
	0.00726965	**27**	109	GO:0016671	oxidoreductase activity, acting on a sulfur group of donors, disulfide as acceptor
	0.00809869	**7**	13	GO:0010291	carotene beta-ring hydroxylase activity
	0.00809869	**12**	33	GO:0016688	L-ascorbate peroxidase activity
	0.00826879	**15**	47	GO:0016168	chlorophyll binding
	0.00848185	**16**	52	GO:0016833	oxo-acid-lyase activity
	0.00893029	**11**	29	GO:0047251;GO:0052639	forming) activity
	0.00952937	**6**	10	GO:0033550	MAP kinase tyrosine phosphatase activity
	0.00952937	**6**	10	GO:0045157	within the noncyclic electron transport pathway of photosynthesis activity
	0.01014909	**83**	463	GO:0016836	hydro-lyase activity
	0.01022678	**16**	53	GO:0004564;GO:0004575	sucrose alpha-glucosidase activity
	0.01080637	**23**	90	GO:0004568	chitinase activity
	0.01179824	**11**	30	GO:0047800	cysteamine dioxygenase activity
	0.01231022	**15**	49	GO:0008121;GO:0016681	ubiquinol-cytochrome-c reductase activity
	0.01231022	**50**	252	GO:0009678	pyrophosphate hydrolysis-driven proton transmembrane transporter activity
	0.01297774	**10**	26	GO:0004347	glucose-6-phosphate isomerase activity
	0.0147231	**40**	192	GO:0003727	single-stranded RNA binding
	0.01500491	**15**	50	GO:0009916	alternative oxidase activity
	0.01500491	**112**	667	GO:0016830	carbon-carbon lyase activity
	0.0152172	**11**	31	GO:0004372	glycine hydroxymethyltransferase activity
	0.01675639	**6**	11	GO:0031409	pigment binding
	0.01755597	**4**	5	GO:0008782	adenosylhomocysteine nucleosidase activity
	0.01755597	**4**	5	GO:0008824	cyanate hydratase activity
	0.01755597	**4**	5	GO:0052692	raffinose alpha-galactosidase activity
	0.01755597	**4**	5	GO:0090409	malonyl-CoA synthetase activity
	0.01771413	**14**	46	GO:0070567	cytidylyltransferase activity
	0.01942572	**5**	8	GO:0004455	ketol-acid reductoisomerase activity
	0.01949864	**100**	591	GO:0030170;GO:0070279	pyridoxal phosphate binding
	0.02080997	**16**	57	GO:0015112	nitrate transmembrane transporter activity
	0.02439254	**32**	149	GO:0016868	phosphotransferases
	0.0249541	**11**	33	GO:0015556;GO:0015140	activity
	0.02731154	**6**	12	GO:0043715;GO:0043716;GO:0046570	methylthioribulose 1-phosphate dehydratase activity
	0.02752666	**761**	5501	GO:0022857;GO:0005215	transporter activity
	0.02797261	**8**	20	GO:0004645	1,4-alpha-oligoglucan phosphorylase activity
	0.0281995	**67**	375	GO:0051287	NAD binding
	0.02852255	**7**	16	GO:0004750	ribulose-phosphate 3-epimerase activity
	0.02852255	**7**	16	GO:0015658	transporter activity
	0.03382836	**9**	25	GO:0003983;GO:0051748	activity
	0.03561154	**5**	9	GO:0010297	heteropolysaccharide binding
	0.04019476	**6**	13	GO:0003827	alpha-1,3-mannosylglycoprotein 2-beta-N-acetylglucosaminyltransferase activity
	0.04019476	**6**	13	GO:0004605	phosphatidate cytidylyltransferase activity
	0.04019476	**6**	13	GO:0004737	pyruvate decarboxylase activity
	0.04019476	**4**	6	GO:0008721;GO:0030378	D-serine ammonia-lyase activity
	0.04019476	**4**	6	GO:0008922	ligase activity
	0.04019476	**7**	17	GO:0009927;GO:0004740	histidine phosphotransfer kinase activity

	0.04019476	**4**	6	GO:0030619	U1 snRNA binding
	0.04019476	**4**	6	GO:0046863	carboxylase/oxygenase activator activity
	0.04019476	**6**	13	GO:0047631	ADP-ribose diphosphatase activity
	0.04245578	**9**	26	GO:0004096	catalase activity
	0.04941245	**73**	426	GO:0016831	carboxy-lyase activity
Cellular Components	5.65E-139	**897**	2987	GO:0009532;GO:0009570	plastid stroma
	2.92E-124	**857**	2933	GO:0009526;GO:0009941	plastid envelope
	6.35E-109	**637**	1986	GO:0009534;GO:0031976;GO:0009579	thylakoid
	2.32E-96	**655**	2195	GO:0042170	plastid membrane
	3.01E-68	**459**	1509	GO:0009535;GO:0055035;GO:0042651;GO:0034357;GO:0044436	obsolete thylakoid part
	2.81E-67	**1051**	4845	GO:0031967;GO:0031975	envelope
	6.10E-42	**656**	2955	GO:0031984	organelle subcompartment
	1.27E-38	**465**	1916	GO:0048046	apoplast
	3.70E-37	**296**	1037	GO:0031969	chloroplast membrane
	3.30E-35	**115**	246	GO:0010287	plastoglobule
	1.35E-29	**143**	390	GO:0009528;GO:0009706	plastid inner membrane
	2.23E-27	**101**	234	GO:0010319	stromule
	4.03E-16	**94**	282	GO:0009543;GO:0031978;GO:0031977	thylakoid lumen
	8.75E-14	**269**	1261	GO:0019866	organelle inner membrane
	5.31E-12	**24**	35	GO:0009509	chromoplast
	2.22E-11	**627**	3621	GO:0005576	extracellular region
	2.92E-05	**14**	25	GO:0000275;GO:0005754;GO:0045267	mitochondrial proton-transporting ATP synthase complex, catalytic sector F(1)
	3.03E-05	**13**	22	GO:0009503;GO:0030076;GO:0009517	light-harvesting complex
	6.28E-05	**44**	159	GO:0009523	photosystem II
	0.00011107	**20**	50	GO:0010598	(plastoquinone)
	0.00012535	**68**	293	GO:0070469	respirasome
	0.00016341	**33**	110	GO:0005747;GO:0045271;GO:0030964	respiratory chain complex I
	0.00028359	**23**	66	GO:0005753	synthase complex
	0.00105664	**53**	227	GO:0005746;GO:0098803	mitochondrial respirasome
	0.00129123	**15**	37	GO:0098807	complex
	0.00134972	**5**	5	GO:0009571	proplastid stroma
	0.00282925	**50**	219	GO:0009521	photosystem
	0.00366541	**13**	32	GO:0009533	chloroplast stromal thylakoid
	0.00923751	**7**	12	GO:0031898;GO:0046862	chromoplast membrane
	0.0138513	**15**	45	GO:0005750;GO:0045275	mitochondrial respiratory chain complex III
	0.01551225	**14**	41	GO:0045261	catalytic core F(1)
	0.01658903	**67**	339	GO:0098800	inner mitochondrial membrane protein complex
	0.01999739	**8**	17	GO:0005846	nuclear cap binding complex
	0.02670134	**7**	14	GO:0010170;GO:1902503	glucose-1-phosphate adenylyltransferase complex
	0.02787907	**23**	89	GO:0009501	amyloplast
	0.02787907	**23**	89	GO:0045259	proton-transporting ATP synthase complex
	0.03729918	**5**	8	GO:0009537	proplastid
	0.04337991	**33**	148	GO:0033178	complex, catalytic domain

Table S6: Comparison of ethylene signaling genes between ECO-R and ECO-S after quinclorac treatment (pvalue < 0.01, FDR < 0.05).

Gene Discription	ECO-R		ECO-S	
	Probe ID generated by Trinity pipeline	Log FC	Probe ID generated by Trinity pipeline	Log FC
1-aminocyclopropane-1-carboxylate oxidase	TRINITY_DN118542_c0_g3_i11	2.01	TRINITY_DN118542_c0_g5_i5	2.01
	TRINITY_DN118542_c0_g3_i13	1.96	TRINITY_DN118542_c0_g5_i1	1.88
	TRINITY_DN118542_c0_g3_i1	1.88	TRINITY_DN112323_c2_g5_i1	2.89
	TRINITY_DN118542_c0_g3_i10	1.54	TRINITY_DN118902_c1_g3_i11	1.70
	TRINITY_DN101948_c1_g1_i5	3.66		
	TRINITY_DN101948_c1_g1_i3	3.65		
	TRINITY_DN101948_c1_g1_i7	3.54		
	TRINITY_DN103345_c0_g4_i2	1.85		
	TRINITY_DN103345_c0_g4_i1	1.70		
	TRINITY_DN108035_c2_g4_i4	3.41		
	TRINITY_DN108035_c2_g4_i2	3.00		
	TRINITY_DN108035_c2_g4_i7	2.67		
	TRINITY_DN108035_c2_g4_i3	2.54		
1-aminocyclopropane-1-carboxylate synthase	TRINITY_DN116903_c0_g1_i1	*1.16	TRINITY_DN116903_c0_g1_i5	-2.09
	TRINITY_DN116903_c0_g1_i10	**1.50	TRINITY_DN116903_c0_g1_i1	-2.20
Ethylene insensitive 3	TRINITY_DN106146_c1_g1_i8	4.36	TRINITY_DN106146_c1_g1_i8	2.23
	TRINITY_DN106146_c1_g1_i7	4.35	TRINITY_DN106146_c1_g1_i1	1.69
	TRINITY_DN106146_c1_g1_i1	4.34	TRINITY_DN106630_c0_g1_i1	-0.85
	TRINITY_DN106146_c1_g1_i3	4.33		
	TRINITY_DN106146_c1_g1_i5	4.02		
	TRINITY_DN106146_c1_g1_i2	3.80		
	TRINITY_DN106146_c1_g1_i4	3.40		
	TRINITY_DN106146_c1_g1_i9	3.35		
	TRINITY_DN123441_c2_g2_i1	3.06		
	TRINITY_DN123441_c2_g2_i3	2.59		
Ethylene-overproduction protein	TRINITY_DN117217_c0_g1_i1	2.89	TRINITY_DN123634_c0_g1_i2	1.22
ethylene-responsive element-binding protein	TRINITY_DN115134_c1_g1_i10	2.68	TRINITY_DN115134_c1_g1_i10	1.25
	TRINITY_DN115134_c1_g1_i6	2.51	TRINITY_DN115134_c1_g1_i6	1.18
Ethylene-responsive protein	TRINITY_DN123585_c1_g3_i1	10.12	TRINITY_DN107218_c1_g1_i4	1.70
	TRINITY_DN123585_c1_g3_i2	9.64	TRINITY_DN107218_c1_g1_i1	1.67
	TRINITY_DN100228_c0_g1_i3	8.41	TRINITY_DN107218_c1_g1_i3	1.67
	TRINITY_DN91246_c0_g1_i2	8.16	TRINITY_DN123585_c1_g3_i2	3.55
	TRINITY_DN118949_c1_g1_i2	7.76	TRINITY_DN123585_c1_g3_i1	3.54
	TRINITY_DN118949_c1_g1_i1	6.57	TRINITY_DN113394_c0_g1_i1	5.90
	TRINITY_DN124268_c2_g1_i2	6.52	TRINITY_DN113394_c0_g1_i4	5.79
	TRINITY_DN113064_c4_g4_i6	6.03	TRINITY_DN113394_c0_g1_i3	5.74
	TRINITY_DN113064_c4_g4_i3	5.92	TRINITY_DN114832_c2_g1_i2	5.49
	TRINITY_DN110715_c3_g1_i3	5.85	TRINITY_DN114832_c2_g1_i1	5.12
	TRINITY_DN124268_c2_g1_i3	5.81	TRINITY_DN114832_c2_g1_i7	4.72
	TRINITY_DN113064_c4_g4_i1	5.63	TRINITY_DN114832_c2_g1_i5	3.98
	TRINITY_DN124268_c2_g1_i1	5.53		
	TRINITY_DN114832_c2_g1_i5	6.36		
	TRINITY_DN114832_c2_g1_i3	5.25		
	TRINITY_DN114832_c2_g1_i8	5.16		
NCED1	TRINITY_DN119516_c0_g1_i6	-3.76	TRINITY_DN119895_c0_g6_i1	4.55
	TRINITY_DN119516_c0_g1_i1	-3.36	TRINITY_DN119895_c0_g6_i11	4.14
			TRINITY_DN119895_c0_g6_i6	4.08
			TRINITY_DN119895_c0_g6_i2	3.47
nitrilase	TRINITY_DN109785_c1_g1_i3	2.91		
nitrilase-associated protein	TRINITY_DN99211_c2_g1_i1	4.14		
	TRINITY_DN99211_c2_g1_i4	2.61		
	TRINITY_DN99211_c2_g1_i3	2.61		
Bifunctional nitrilase/nitrile hydratase	TRINITY_DN108476_c2_g1_i4	1.14		
	TRINITY_DN108476_c2_g1_i5	1.12		
*pvalue 0.07<br>**pvalue 0.08				

Table S7: List of genes upregulated in ECO-R after quinclorac treatment (pvalue < 0.01, FDR < 0.05).

Category	Description of gene	No. of Transcripts	Av. log FC
**Ascorbate recycling mediated ROS activities**	bifunctional monodehydroascorbate reductase and carbonic anhydrase	3	1.6
	dehydroascorbate (DHA) reductase	4	2.4
	Glutathione reductase	2	2
	L-ascorbate oxidase homolog	4	3
	Monodehydroascorbate reductase	4	2
**Glutathiol-mediated and Thioredoxin mediated ROS activities**	Glutaredoxin (GRXC1)	1	8.4
	Glutaredoxin (GRXS5)	2	1.7
	Glutaredoxin (UNKNOWN)	4	1.5
	Glutaredoxin (Y5986)	2	2
	glutathione peroxidase	3	2.8
	glutathione s-transferase (GSTU1)	2	1.2
	glutathione s-transferase (Unknown)	20	2.5
	glutathione s-transferase (GSTU6)	5	2.5
	Thioredoxin (TRXM2)	1	2.1
	Thioredoxin (TRH42)	3	1.8
	Thioredoxin (TRL11)	8	2.8
	Thioredoxin (TRXM)	1	1.9
	Thioredoxin (TRXM1)	2	2.2
	Thioredoxin (UNKNOWN)	6	1.5
	Peroxiredoxin	5	2
**Raffinose synthesis**	Raffinose synthase 6	3	1.9
	Raffinose synthase 2	10	3.3
**Vitamin E biosynthesis**	2-methyl-6-phytyl-1,4-hydroquinone methyltransferase	5	1.5
	4-hydroxyphenylpyruvate dioxygenase	2	2
	Aminotransferase	5	1.2
	fat-soluble vitamin metabolic process	3	2.2
	gamma-tocopherol methyltransferase	4	1.2
	geranylgeranyl reductase	1	2.4
	Homogentisate phytyltransferase	5	1.8
	Phytyl-phosphate kinase (VTE6)	2	1.5
	tocopherol cyclase	2	2.3
**Trehalose Pathway**	trehalose-phosphate phosphatase (unknown)	7	3.8
	trehalose-phosphate phosphatase 7	2	2.5
	trehalose-phosphate phosphatase E	1	2.3
	trehalose-phosphate phosphatase J	1	2.1

	Trehalose synthase 1	2	2.1
	Trehalose synthase 5	3	3.2
	Trehalose synthase 6	3	2.8
	Trehalose synthase 7	11	2.5
	Trehalose synthase 9	1	4.5
	Trehalose synthase 5	6	3.5
**Cellular import of allantoin and allantoic acid**	Ureide permease	1	3.6
**Proline Accumulation**	Ornithine aminotransferase (OAT)	5	2.4
	Delta-1-pyrroline-5-carboxylate synthase (P5CS)	2	2
**Rhamnose Synthesis**	Rhamnose biosynthetic enzyme (RMLCD)	6	5.6

Table S8: Treatment structure for for the RNA-seq experiment.

Accession	Treatment	Herbicide	Application Rate	Adjuvant	Adjuvant Concentration
ECO-R	treated	Quinclorac	560 g ha^{-1}	Crop oil	1% v/v
	nontreated	None	-	-	-
ECO-S	treated	Quinclorac	560 g ha^{-1}	Crop oil	1% v/v
	nontreated	None	-	-	-

Table S9: RNA primers used for the qRT-PCR gene expression assay with and without quinclorac treatment.

Gene	Fwd/ Rev	Sequence (5’ to 3’)
TPP	Forward	CACCTGACGAAGATCCTGCT
	Reverse	ATCTTCCTGCCTTGTGCATT
TPS6	Forward	AACGATCGAGGACAAGGAGA
	Reverse	GACGCTCTCCAGATGGTCA
UGT75D1	Forward	GCTCACTTTCCCGTTCCAG
	Reverse	GTGGTGGAGAATGTGACGAG
UGT73D1	Forward	GTGAACACGTTCCTGGACCT
	Reverse	TCGACGTCTTTGTTCACGAG
CYP709B1	Forward	GTCGTCAAGCAGGTGCTCTT
	Reverse	CAGTGAGGACGAGACCCTTG
CYP709B2	Forward	GCCTGAGAGGTTCGAGTACG
	Reverse	CGATCATCGCAAAGTTCTGA
UGT73E1	Forward	TCTCCGACCAGTTCCTCAAC
	Reverse	CATCGTCCAGCATGTACACC
TIR1	Forward	TGGAGAAATGCAAAATGCTG
	Reverse	GGCCTGTTAGGTGGTGACAG

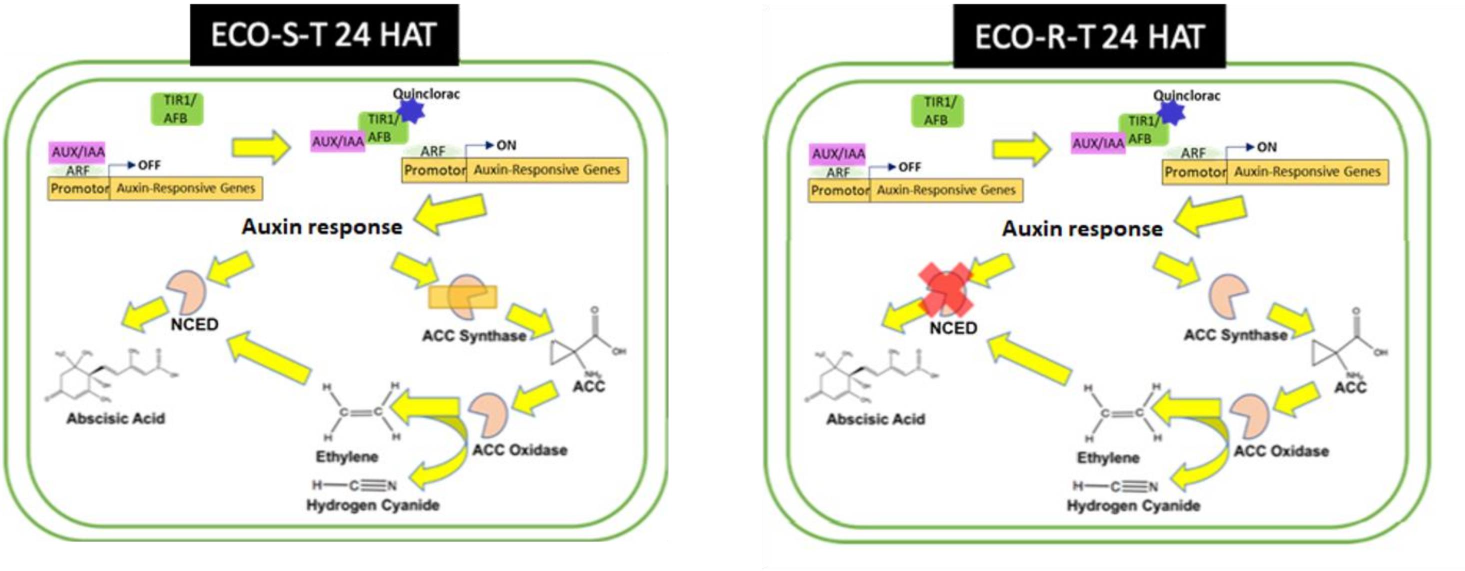

Figure S1: Diagram depicting the quinclorac-activated physiological pathway as explained in the literature [21] and the response of ECO-S and ECO-R, 24 h after treatment, as deduced by the transcriptome sequence analysis. Orange negative (-) symbols indicate repressed enzyme transcripts, green plus symbols (+) indicate induced enzyme transcripts, and red crosses (X) indicate highly repressed transcripts.
